# Making Drug Approval Decisions in the Face of Uncertainty: Cumulative Evidence versus Value of Information

**DOI:** 10.1177/0272989X241255047

**Published:** 2024-06-03

**Authors:** Stijntje W. Dijk, Eline Krijkamp, Natalia Kunst, Jeremy A. Labrecque, Cary P. Gross, Aradhana Pandit, Chia-Ping Lu, Loes E. Visser, John B. Wong, M. G. Myriam Hunink

**Affiliations:** Department of Epidemiology, Erasmus University Medical Center, Rotterdam, The Netherlands; Department of Radiology and Nuclear Medicine, Erasmus University Medical Center, Rotterdam, The Netherlands; Erasmus School of Health Policy & Management, Erasmus University Rotterdam, Rotterdam, The Netherlands; Centre for Health Economics, University of York, York, UK; Cancer Outcomes, Public Policy, and Effectiveness Research (COPPER) Center, Yale University School of Medicine, New Haven, CT, USA; Department of Epidemiology, Erasmus University Medical Center, Rotterdam, The Netherlands; Cancer Outcomes, Public Policy, and Effectiveness Research (COPPER) Center, Yale University School of Medicine, New Haven, CT, USA; Department of Epidemiology, Erasmus University Medical Center, Rotterdam, The Netherlands; Department of Epidemiology, Erasmus University Medical Center, Rotterdam, The Netherlands; Department of Hospital Pharmacy, Erasmus University Medical Center, Rotterdam, The Netherlands; Hospital Pharmacy, Haga Teaching Hospital, The Hague, The Netherlands; Division of Clinical Decision Making, Tufts Medical Center, Boston, USA; Department of Epidemiology, Erasmus University Medical Center, Rotterdam, The Netherlands; Department of Radiology and Nuclear Medicine, Erasmus University Medical Center, Rotterdam, The Netherlands; Center for Health Decision Science, Harvard T.H. Chan School of Public Health, Boston, MA, USA

**Keywords:** antibodies, cost-benefit analysis, COVID-19, decision support techniques, drug approval, meta-analysis, monoclonal, policy analyses

## Abstract

**Background:**

The COVID-19 pandemic underscored the criticality and complexity of decision making for novel treatment approval and further research. Our study aims to assess potential decision-making methodologies, an evaluation vital for refining future public health crisis responses.

**Methods:**

We compared 4 decision-making approaches to drug approval and research: the Food and Drug Administration’s policy decisions, cumulative meta-analysis, a prospective value-of-information (VOI) approach (using information available at the time of decision), and a reference standard (retrospective VOI analysis using information available in hindsight). Possible decisions were to reject, accept, provide emergency use authorization, or allow access to new therapies only in research settings. We used monoclonal antibodies provided to hospitalized COVID-19 patients as a case study, examining the evidence from September 2020 to December 2021 and focusing on each method’s capacity to optimize health outcomes and resource allocation.

**Results:**

Our findings indicate a notable discrepancy between policy decisions and the reference standard retrospective VOI approach with expected losses up to $269 billion USD, suggesting suboptimal resource use during the wait for emergency use authorization. Relying solely on cumulative meta-analysis for decision making results in the largest expected loss, while the policy approach showed a loss up to $16 billion and the prospective VOI approach presented the least loss (up to $2 billion).

**Conclusion:**

Our research suggests that incorporating VOI analysis may be particularly useful for research prioritization and treatment implementation decisions during pandemics. While the prospective VOI approach was favored in this case study, further studies should validate the ideal decision-making method across various contexts. This study’s findings not only enhance our understanding of decision-making strategies during a health crisis but also provide a potential framework for future pandemic responses.

**Highlights:**

- This study reviews discrepancies between a reference standard (retrospective VOI, using hindsight information) and 3 conceivable real-time approaches to research-treatment decisions during a pandemic, suggesting suboptimal use of resources.
- Of all prospective decision-making approaches considered, VOI closely mirrored the reference standard, yielding the least expected value loss across our study timeline.
- This study illustrates the possible benefit of VOI results and the need for evidence accumulation accompanied by modeling in health technology assessment for emerging therapies.

## Introduction

Facing the difficult task of approving new therapies, policy makers often find themselves navigating complex choices under time pressure and limited evidence.^1^ When new information emerges on the clinical effectiveness of treatment options, they can decide to outright approve or reject potential treatments. Alternatively, they may also opt for demanding further trials, thereby delaying the approval process (usage only in research settings [OIR] or Investigational New Drug status), or grant emergency use authorization (EUA) while still conducting research (approval with research; AWR).^1,2^ Balancing these choices has high stakes, in which the aim is to weigh maximizing certainty about treatment effectiveness against the risks of delay or implementing ineffective treatments.^3^

The COVID-19 pandemic vividly underscored these challenges. When the pandemic was declared in 2020, there were no specific treatments available to treat patients affected by the SARS-CoV-2 virus. Early guidelines relied heavily on expert opinions and experiences from previous pandemics. Meanwhile, each decision delay affected thousands of lives, with 3,000 to 5,500 daily hospitalizations in the United States alone.^4^

The toolset available for making such pivotal decisions is multifaceted. Policy makers may lean on individual trials for guidance or, when multiple trials have been conducted, aggregate the available evidence through meta-analyses. These methods, despite being common practice, often hinge on statistical significance. They frequently suggest further research is needed^5^ but fall short in quantifying decision-making consequences. An alternative approach is to use value-of-information (VOI) analyses. This decision-analytic framework accounts for current uncertainties tied to the decision and forecasts the potential implications of deciding on approval with and without further research.^5–9^ VOI presents a more holistic perspective, contrasting the potential benefits of more research against possible lost opportunities due to delays and research costs.^5^

In this study, we identify decisions drawn from each of these approaches across the COVID-19 timeline, including policy decisions as they were made, decisions suggested by cumulative meta-analysis (CMA), and decisions informed by prospective VOI with information available then. We then compare these to a reference standard, represented by the VOI-based approach equipped with the benefit of hindsight (retrospective). We apply these methods to the example of monoclonal antibodies (MAbs), which are designed to mimic the body’s natural immune response, in the treatment of hospitalized COVID-19 patients.^10^

## Methods

We compared 3 distinct approaches to guide policy makers in their choices using an example of the recommendations on implementation and further research of treatment with MAbs to usual care for hospitalized COVID-19 patients across the timeline of accrual of evidence:

1. **The optimal strategy based on policy recommendations on drug approval**. Here we use the decisions on EUA or approval made by the US Food and Drug Administration (FDA). We consider the strategy the FDA implemented to be the optimal strategy from their perspective.
2. **The optimal strategy based on CMA.** In this approach, the decision is based solely on the rolling result of the CMA. A mortality reduction with a significance level *P* ≤ 0.05 leads to the decision “approve” being considered as optimal by the CMA and *P* > 0.05 to “AWR,” while an increase in mortality with a significance level *P* > 0.05 leads to the decision “OIR” and *P* ≤ 0.05 to “reject.”
3. **The optimal strategy based on prospective decision-analytic and VOI results.** In this approach, we use only information available at the time point of the decision. The optimal strategy is the strategy for which the VOI identifies the highest expected value.

In our analysis, we assumed that the retrospective decision-analytic and VOI results (with information available in hindsight) represent the reference standard and that the optimal strategy identified with this approach is the *true* optimal decision. This optimal strategy is the strategy that yielded the highest expected value (EV), given that we now know how many patients would truly be affected by the decision throughout the pandemic. Thus, we used this fourth approach as a comparator to evaluate the results of each of the 3 examined approaches. By comparing the EV of the suggested strategy at that time for each approach to the true optimal strategy, we estimate the value loss.

The sections below provide methodological details on each of the evaluated approaches. Publications are referred to in the analysis and figures by their respective trial names or, if no trial name was assigned, by the first author’s name.

### Approach 1: Policy Recommendations on Drug Approval

To evaluate the first approach, which proposes the optimal strategy is represented by the actual policy decision, we reviewed the press releases by the FDA for information on the EUA or approval of MAbs in the treatment of COVID-19.

### Approach 2: CMA

To evaluate the effectiveness of the second approach, which proposes to base the decision making on CMA, we performed a systematic review and CMA of all randomized controlled trials (RCTs) investigating MAbs. Our objective is to identify the relative risk of mortality at day 28 of MAbs versus usual care in hospitalized COVID-19 patients.

#### Eligibility criteria

- Population: The study population included hospitalized adult (≥18 y) COVID-19 patients.
- Intervention: The intervention was treatment with any form of Mabs.
- Control: The study included usual care as the control arm condition.
- Outcome: Study outcomes reported mortality at 1 mo (28 d to 31 d after Rx).
- Study design: The study was an RCT published in English between December 2019 and June 2021.

#### Search strategy

The literature search was conducted and optimized with the help of an information specialist from the Erasmus MC Medical Library. Citations were identified from 6 databases:

- Excerpta Medica dataBASE (EMBASE)
- Medical Literature Analysis and Retrieval System Online (MEDLINE) via OVID
- Cochrane Central Register of Controlled Trials
- Web of Science core collection
- World Health Organization COVID-19 database
- Google scholar

We reviewed the references of systematic reviews identified by the search for additional citations. The full search strategy is provided in Appendix Tables 1 and 2. We followed the PRISMA reporting recommendations.^11^

#### Study selection, data extraction, and bias assessment

Two researchers independently screened articles based on title and abstract and full text and performed data extraction. The Rayyan QCRI data management tool was used to store, organize, and manage all references.^12^ Discrepancies were resolved by consensus or with a third reviewer. We assessed the quality of included studies using the Cochrane Risk of Bias-2 tool^13^ and reviewed the presence of publication bias through a Funnel plot.

#### Publication date

The publication date is noted as the first date the article became available online as a preprint (identified on medRXiv or Research Square using the trial ID) or published in a peer-reviewed journal. We assume this date is most in line with when the information became available to decision makers. We justified this choice based on previous studies that concluded that the main RCT results were consistent between preprint and printed journal articles and because some articles were delayed in publication.^14^ As our search strategy included trial registers, studies that were published beyond the last search date (June 2021) were eligible for inclusion if they were identified through the initial search.

#### Statistical analysis

The effect estimate for mortality was expressed as the relative risk (RR) of dying in the intervention arm compared with the control arm. We assume that, despite the diverse biological pathways targeted by MAbs, their mechanism of action is homogenous enough for meaningful meta-analysis. We performed a CMA of all included articles using a random-effects model.^15^ We used the Mantel-Haenszel method for pooling and quantifying expected heterogeneity with an I^2^ statistic. Between-study variance was quantified using the tau^2^ statistic, and its uncertainty was adjusted using the Knapp-Hartung adjustment.^16^

In addition, we performed a meta-regression with severity as a predictor using articles that reported sufficient information. For this analysis, patients not on supplemental oxygen or oxygen delivery by face mask/nasal cannula were deemed nonsevere and assumed to be treated in the ward, while patients on noninvasive ventilation, invasive mechanical ventilation, or extracorporeal membrane oxygenation were categorized as severe and assumed to be treated in an intensive care unit (ICU) setting.

All statistical analyses were performed with R (version 4.1.2.).^17^

### Approach 3: Decision-Analytic Model and VOI Analysis

#### Model description

To evaluate the third approach (i.e., prospective VOI that proposes to base decision making on decision-analytic methods), we used a decision-analytic model and performed VOI analyses.

The state-transition cohort model consists of 4 health states: 1) hospitalized, 2) recovered from the ICU as the highest level of care, 3) recovered from the hospital ward as the highest level of care, 4) dead. The model structure is provided in Appendix Figure 7, and further details on this decision-analytic model are available in Dijk et al.^3^ The model was developed based on the DARTH framework.^18–20^ We followed the CHEERS,^21^ CHEERS-VOI,^22^ and ISPOR^23^ reporting recommendations.

Whenever a new study is incorporated into the CMA, the decision model recalculates the pooled effect. Based on these updated data, it then estimates incremental cost-effectiveness ratios of treatment with the drug versus usual care without the drug.

All other model parameters were based on best available evidence as of December 2021. An overview of these parameters is provided in the supplementary materials (Supplementary File: Excel). We applied the cost and length of stay of tocilizumab as the most frequently used drug in the meta-analysis as the cost of treatment. Our analysis was conducted from the US health-system perspective with a $100,000/quality-adjusted life-year (QALY) willingness-to-pay (WTP) threshold^24–27^ with a lifetime horizon. Costs were estimated in 2020 US dollars ($). We applied a 3% annual discount rate for both costs and effects, and probabilistic analysis was performed with 10,000 iterations.

A VOI analysis is a decision-analytic method that helps assess and quantify decision uncertainty and determine whether the available evidence is sufficient to make an immediate decision or if further research is needed.^28^ For the purpose of our evaluation, we calculated the expected added value of performing an RCT to reduce the uncertainty surrounding the treatment effect on mortality as partial perfect information (EVPPI) using a linear-regression meta-model.^29^ For time points with a positive value of further research (EVPPI > 0), we performed an expected value of sample information (EVSI) estimation using a Gaussian approximation approach as proposed by Jalal and Alarid-Escudero.^30–32^ We next identified the optimal sample size of a new RCT. Further details on VOI methodology is provided in the published literature,^1,2,5,28,33–35^ including in the context of COVID-19.^3^

#### Prospective VOI analysis

The prospective VOI approach mimics a situation in which we had used VOI to inform decisions across the timeline. This analysis uses the number of patients affected by the decision based on the available information at that time point. In other words, the analysis presents the results we would have received at that time.

This analysis uses the sum of the number of daily hospitalizations forecasted by the Institute for Health Metrics and Evaluation (IHME)^36,37^ as of the latest available data set at the time of publication of the last study added to the CMA until the end of the forecast (Appendix Figure 9). When IHME predictions ended mid-peak, we extended these predictions under the assumption that peaks during a pandemic have symmetric inclines and declines, which would be more realistic than a sudden drop in patients at those time points. A depiction of the unextended and extended predictions can be found in Appendix Figure 10.

The net benefit obtained with EUA of treatments while performing further RCTs was determined for the expected number hospitalizations in the United States while awaiting trial results and their implementation (current patients) over 2 mo. We chose 2 mo based on the availability of predictions: a minimum of half of the prediction time needs to contain predictions on future patients. We calculated our results for a maximum feasible sample size of 2,500 patients (main analyses) and the optimal sample size capped at 10,000.

**Figure 1:**
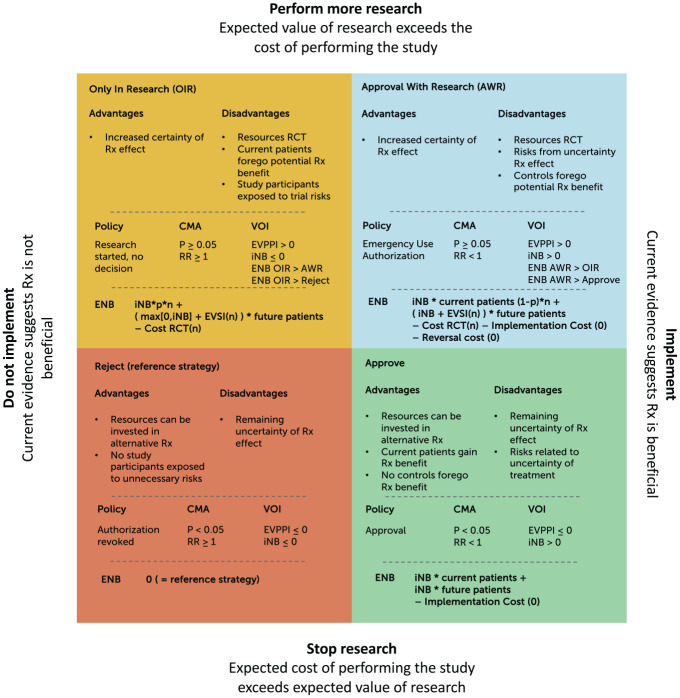
Decision matrix. This 2 × 2 figure shows the 4 potential combined research-treatment strategies, their advantages and disadvantages, the decision rule when this strategy would be selected as the optimal strategy, and how the expected net monetary benefit (ENB) would be calculated for the value-of-information (VOI) analyses to determine the optimal strategy according to the VOI. Difference retrospective/prospective VOI: the number of patients is based on the projections on the date of evaluation (prospective) or on the last data set on the timeline (retrospective). iNB, incremental net (monetary) benefit (NB treat – NB control); p, proportion of patients *n* in a new randomized controlled trial (RCT) randomly assigned to treatment; 1 − p, proportion of patients *n* in a new RCT randomly assigned to control; EVSI, expected value of sample information, calculated in comparison with the optimal treatment (i.e., over and above the iNB gained if iNB > 0); EVSI(*n*), EVSI dependent on sample size *n* of the new RCT; cost RCT(*n*), fixed cost + variable cost of performing a new RCT dependent on sample size *n*. The optimal sample size is determined by maximizing the function in the quadrant with respect to *n*. Implementation and reversal costs are assumed to be 0 given that the intervention concerns a guideline change.

The net value of each strategy was calculated according to the equations listed in Figure 1.

#### Reference standard: retrospective VOI analysis

The reference standard, also referred to as the retrospective VOI, mimics a situation in which we compare VOI analysis to make decisions at various time points across a timeline with hindsight knowledge. Namely, in this analysis, we use the sum of the number of daily hospitalizations observed and forecasted in the IHME data set of the last date prior to the end of our timeline (December 2021)^37^ (Appendix Figure 8). We consider retrospective VOI as the reference standard since it contains the most information and considers both uncertainty and the consequences of making a suboptimal decision using information in hindsight.

#### Sensitivity analyses

We provide additional cost-effectiveness acceptability curves across time points illustrating how the EVPPI results are influenced by the chosen WTP threshold. We performed several scenario analyses, in which we

- increased the WTP threshold to $150,000,
- increased the trial duration from a 2-mo to a 3-mo period,
- included only the 4 trials listed in the FDA EUA press release,
- did not extrapolate data sets that ended in peak predictions,
- use noncumulative individual study data,
- conduct the meta-analysis without the Knapp-Hartung adjustment,
- conduct the meta-analysis based on a subgroup of only interleukin (IL)–6-(R-) inhibitor MAbs. We performed a subgroup analysis for this set of MAbs with a similar working mechanism as 16 articles that were included and could be meta-analyzed. Other MAbs included in our study were investigated only in single trials, for which the results can be found in the noncumulative individual study data scenario.

### EV Loss due to Suboptimal Choice

Finally, we calculated the loss in EV for each of the possible approaches. We calculated the expected loss of the strategies proposed by each approach (policy, CMA, or prospective VOI) by comparing their EVs to the EVs of the retrospective VOI (i.e., reference standard). For example, if CMA suggests AWR as the optimal strategy and retrospective VOI suggests approve, then we calculate the net value of the retrospective VOI EV for approve minus the retrospective VOI EV for AWR.

## Results

### Approach 1: Policy Recommendations on Drug Approval

The FDA announced EUA on June 24, 2021,^38^ for tocilizumab, a MAb that reduces inflammation by blocking the IL-6 receptor. The press release states that 4 clinical trials contributed to this decision: RECOVERY,^39^ EMPACTA,^40^ COVACTA,^41^ and REMDACTA.^42^ The EUA statement notes that the known and potential benefits of this drug outweigh the known and potential risks of this treatment. The announcement does not specify the methodological approach to combining or comparing the results of these 4 trials but notes that RECOVERY and EMPACTA provided the most important scientific evidence on the potential benefit. On December 21, 2022, tocilizumab was approved^43^; however, this date falls beyond the timeline of our study.

### Approach 2: CMA

Our search identified 10,633 articles, of which 18 entailing 22 treatment comparisons and 10,031 patients were included in the meta-analysis.^39–41,44–58^ A flowchart detailing the search results is shown in Appendix Figure 1. Study characteristics and quality assessment, a funnel plot, and a global distribution of included studies and meta-regression based on severity can be found in Appendix Tables 3 and 4 and in Appendix Figures 3 and 6.

Ten of the included studies investigated the use of tocilizumab, whereas the remaining studies investigated the use of sarilumab, vilobelimab, itolizumab, mavrilimumab, otilimab, levilimab, siltuximab, and lenzilumab. The total number of events (deaths) was 2,368, of which 1,097 occurred in the intervention group and 1,271 in the control group. The largest study was the RECOVERY trial^39^ with 4,116 subjects (41% of the total), and the smallest study was PANAMO (30 subjects).^44^ Seven studies had fewer than 10 deaths in the intervention arm, and 10 studies had fewer than 10 deaths in the control arm.

**Figure 2:**
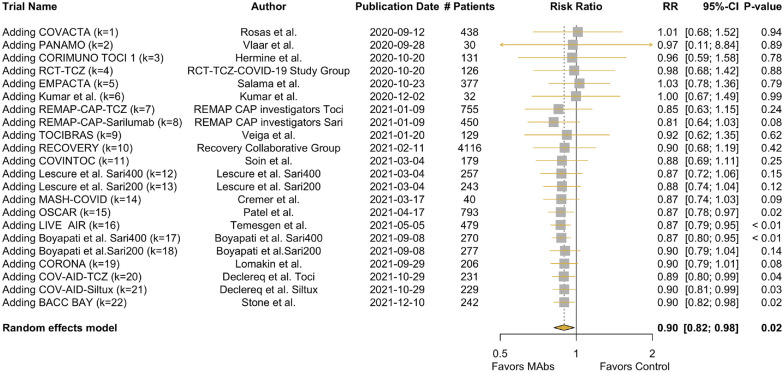
Forest plot of the cumulative meta-analysis. Pooled relative risk for mortality with treatment with monoclonal antibodies versus control arms. At each time point the trial results were published: the relative risk is a pooled result of the newly included study and the evidence thus far accrued. K, number of included studies; Sari, sarilumab; TCZ/Toci, tocilizumab.

Figure 2 shows the results of the CMA in a forest plot, with an overall RR of 0.90 (95% confidence interval 0.82, 0.98) after all studies were included. The traditional (noncumulative) meta-analysis is provided in Appendix Figure 4.

### Approaches 3 and 4: Decision-Analytic Model and VOI Analysis

**Figure 3:**
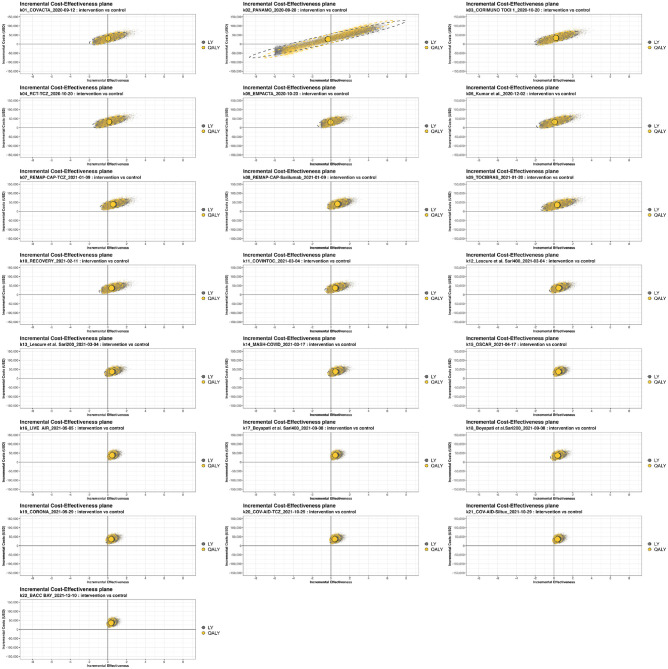
Incremental cost-effectiveness planes of the monoclonal antibody (MAb) treatment versus usual care over time. The *x*-axes show incremental effectiveness, whereas the *y*-axes show incremental costs in USD. This grid slot shows the cost-effectiveness (CE) plot at each time point at which a new study (k) is added to the cumulative meta-analysis treatment effectiveness estimate. The plots are based on the results of 10,000 iterations. The yellow circle depicts the estimate for quality-adjusted life-years (QALYs) and the gray for LY. The dotted line surrounding the mean estimate reflects the uncertainty. Other parameter inputs remained constant over time.

Figure 3 shows the cost-effectiveness plane over time. After an initial increase in uncertainty once the results from the second trial (PANAMO^44^) were added, the uncertainty surrounding the estimated cost-effectiveness increases then decreases again with accrual of further evidence. The figure illustrates that for most time points, the mean and majority of iterations fall in the upper right quadrant, in which both QALYs and costs are higher for MAbs.

Results of the cost-effectiveness analysis (CEA), probabilistic analysis, prospective and retrospective VOI, parameters for the ENB calculations, and the ENB results of each potential strategy are provided in Table 1.

**Table 1:** Summary of Key Calculated Parameters and Results of the CEA, Prospective VOI, and Retrospective VOI^a^

Study Number Added	K01	K02	K03	K04	K05	K06	K07	K08	K09	K10	K11
Trial Name	COVACTA	PANAMO	CORIMUNO TOCI 1	RCT-TCZ	EMPACTA	Kumar et al.	REMAP-CAP-TCZ	REMAP-CAP-Sarilumab	TOBRICAS	RECOVERY	COVINTOC
Main analysis											
Is treatment cost-effective	No	No*	No	No	No*	No	Yes	Yes	No	Yes	Yes
Incremental costs, $)	31,319	26,577	33,659	32,770	30,291	31,804	39,009	40,873	35,665	36,572	37,476
Incremental QALYs	0.03	−0.31	0.19	0.13	−0.04	0.06	0.56	0.69	0.33	0.39	0.45
ICER, $/QALY	1,126,586	n/a	177,503	256,841	n/a	519,873	69,978	59,607	108,972	93,945	83,033
Incremental net monetary benefit, $ (thousand)	−29	−57	−15	−20	−35	−26	17	28	−3	2	8
Prospective VOI analysis											
Current patients (thousand)	290	253	245	245	274	400	530	530	400	324	156
Future patients (thousand)	690	915	708	708	663	273	139	139	62	190	31
popEVPPI, $ (million)	8,542	69,270	15,235	9,304	3,354	3,479	1,430	620	1,266	2,910	330
Optimal strategy	OIR	OIR	OIR	OIR	OIR	OIR	AWR	AWR	OIR	AWR	AWR
Net value, $ (million)	7,371	62,799	13,212	7,588	2,080	2,471	12,029	18,709	839	2,599	1,517
Retrospective VOI analysis											
Current patients (thousand)	331	462	673	673	702	913	579	579	462	337	316
Future patients (thousand)	4,426	4,242	3,933	3,933	3,887	3,288	3,043	3,043	2,991	2,872	2,757
popEVPPI, $ (million)	54,781	321,051	84,567	51,647	19,668	41,848	31,267	13,551	61,134	43,914	29,021
Optimal strategy	OIR	OIR	OIR	OIR	OIR	OIR	AWR	AWR	OIR	AWR	AWR
Net value ($, million)	47,637	291,437	73,571	42,377	12,562	30,436	79,682	105,238	42,188	28,974	34,411

Study Number Added	K12	K13	K14	K15	K16	K17	K18	K19	K20	K21	K22
Trial Name	Lescure et al. Sari400	Lescure et al. Sari200	MASH-COVID	OSCAR	LIVE AIR	Boyapati et al. Sari400	Boyapati et al. Sari200	CORONA	COV-AID-TCZ	COV-AID-Siltux	BACC BAY
Main analysis											
Is treatment cost-effective	Yes	Yes	Yes	Yes	Yes	Yes	Yes	Yes	Yes	No	No
Incremental costs, $	37,899	37,350	37,839	37,687	37,625	37,625	36,325	36,246	36,707	36,186	36,154
Incremental QALYs	0.48	0.44	0.48	0.47	0.46	0.46	0.37	0.37	0.40	0.36	0.36
ICER, $/QALY	78,900	84,441	79,482	81,010	81,647	81,647	97,785	99,084	92,330	100,084	100,628
Incremental net monetary benefit, $ (thousand)	10	7	10	9	8	8	0.8	0.3	3	−0.03	−0.2
Prospective VOI analysis											
Current patients (thousand)	156	156	389	388	336	252	252	462	387	387	441
Future patients (thousand)	31	31	63	38	58	62	62	27	1,054	1,054	275
popEVPPI, $ (million)	247	241	420	161	203	215	568	212	6,609	7,438	1,771
Optimal strategy	AWR	AWR	AWR	Approve	Approve	Approve	AWR	AWR	AWR	OIR	OIR
Net value, $ (million)	1,926	1,329	4,479	3,762	3,339	2,650	474	226	6,610	3,315	743
Retrospective VOI analysis											
Current patients (thousand)	316	316	303	221	165	460	460	382	470	470	893
Future patients (thousand)	2,757	2,757	2,705	2,625	2,590	1,584	1,584	1,456	1,178	1,178	492
popEVPPI, $ (million)	21,735	21,177	18,070	11,207	9,004	5,507	14,558	11,549	7,391	8,318	3,168
Optimal strategy	AWR	AWR	AWR	AWR	AWR	AWR	AWR	AWR	AWR	OIR	OIR
Net value, $ (million)	37,460	28,177	34,105	27,208	24,643	18,062	8,043	5,755	7,507	3,711	1,355

AWR, approval with research; CEA, cost-effectiveness analysis; ICER, incremental cost-effectiveness ratio; n/a, ICER is not applicable because of dominance; OIR, only in research; popEVPPI, population expected value of partially perfect information; QALY, quality-adjusted life-year; VOI, value of information; WTP, willingness to pay.

a Results shown are the mean results from the probabilistic analysis, calculated as the treatment arm versus the usual care arm of each trial added. K represents how many trials have been added into the analysis thus far. Results are presented for QALYs. Yes* = treatment is dominant; Yes = treatment is effective and ICER < WTP; No* = treatment is cost-saving but not enough that ICER > WTP (i.e., treatment is not decrementally cost-effective); Results are rounded off. Future/current patients are based on all expected hospitalized patients. Sample size in the net benefit calculations is used as the optimal sample size or the maximum feasible sample size of *N* = 2,500.

Supportive figures are provided in the appendix, including CE planes (Appendix Figures 11 and 12), CEA curves and frontiers (Appendix Figure 13), expected value of partial sample information plots (Appendix Figure 14), and full results overview for all strategies and including optimal sample size (Appendix Figures 15 and 16).

### Timeline of Approaches to Decision Making

**Figure 4:**
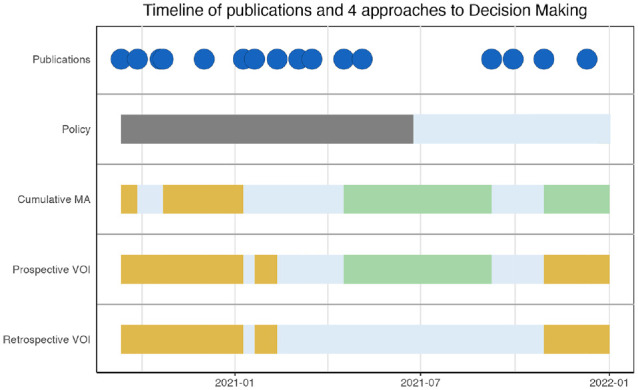
Timeline of publications and 4 approaches to decision making. The plot represents the publication of the results of each study and the optimal strategy as suggested by the 4 approaches (cumulative meta-analysis, policy set by the Food and Drug Administration, prospective value of information [VOI] and retrospective VOI).

We summarized publication dates and the optimal strategies as suggested by each of the approaches in Figure 4. Included articles were published between September 2020 and December 2021.

The first policy decision (EUA) was made in June 2021 after 18 of 22 treatment comparisons from this article were included. Drug approval took place in December 2022, after the last article of our meta-analysis was included, and is therefore not shown in this figure. The CMA suggested AWR earlier and more frequently than prospective or retrospective VOI. Both CMA and prospective VOI suggest approve between the 15th and 18th treatment comparison. The final suggested strategy based on the CMA is approve, in contrast to prospective and retrospective VOIs, which suggest OIR. Retrospective VOI suggests OIR and AWR strategies across the entire timeline but never approval. None of the studies suggest outright rejection.

**Figure 5:**
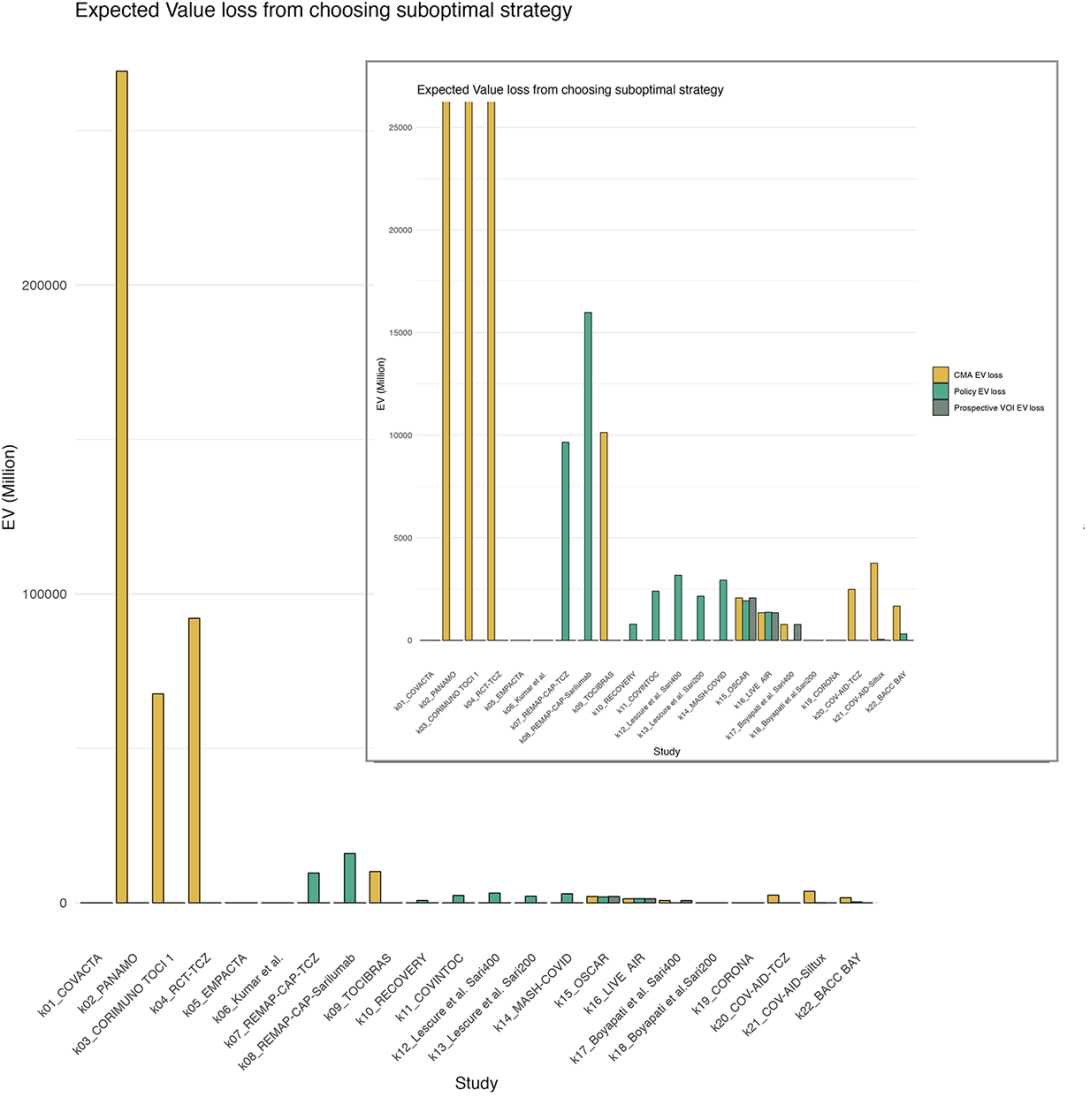
Expected value loss from choosing the suboptimal strategy according to the gold standard compared with the other approaches. This is calculated by taking the EV of the optimal strategy—the EV of the strategy suggested by each respective approach (policy, CMA, or prospective VOI). The EV is provided by the gold standard, as this approach is closest to knowing the true EV as the largest proportion of hospitalizations have already been observed. The smaller figure in the top right corner is the same plot with a zoomed-in version of the plot with a *y*-axis set to max $25,000 million. The highest losses for each strategy were CMA approach: $269 billion; policy approach: $16 billion; prospective VOI approach: $2 billion. CMA, cumulative meta-analysis; EV, expected value; VOI, value of information.

In Figure 5, we have depicted the loss that would result from a suboptimal choice in strategy in comparison with the reference standard. This loss is calculated based on the values as presented in Appendix Figure 16. Figure 5 shows that the expected loss is highest when decision making is guided by CMA alone, particularly in early stages (k2-k4). Here, CMA suggests AWR while prospective and retrospective VOIs suggest that OIR is the optimal strategy (Box 1).

**Box 1:** Example of Reading the Results for 1 Time Point (January 9, 2021)

Example Suppose it is January 9, 2021, and the results of the REMAP-CAP study have just been published (k7 and k8). Figure 4 tells us that at this time, no emergency approval was provided by the FDA, and hence they consider it applicable only in research settings. The cumulative meta-analysis finds that when pooling the results from all studies until k8, with in total 2,339 people included in these studies, an RR 0.81 [0.64;1.03], *P* = 0.08, to die with treatment at 28 d (Figure 2). This suggests AWR is the optimal strategy. The decision model finds the treatment is cost-effective (ICER $59,607/QALY; Table 1). At this time, we expect that 139,000 more people will be admitted in the upcoming 2 mo and 530,000 in the months thereafter who can benefit from our decision. Prospective VOI suggests that AWR is the optimal strategy, with a new study with a sample size of 6,900. Capping our max sample size at 2,500, a new trial costs $32 million, with an expected benefit of $18,709 million (Appendix Figure 15). Retrospective VOI shows us that, in fact, 3,043,000 were admitted in the 2 mo after REMAP-CAP’s publication and an additional 579,000 until the end of our timeline. Retrospective VOI also suggests AWR is the optimal strategy, but a higher sample size of >10,000 is considered optimal. Capped at a sample size of 2,500, the expected value of AWR is $105,238 million (Appendix Figure 16). On this date, CMA, prospective VOI, and retrospective VOI agreed on the optimal strategy being AWR. The policy approach, however, suggested OIR. Based on retrospective VOI, the expected value of OIR at this time point is $89,257 million, which means an expected loss of $105,238M to $89,257M = $15,981M if we relied on the policy approach (Figure 5, Appendix Figure 16) and a loss of $ 0 if we used CMA or prospective VOI.

AWR, approval with research; CMA, cumulative meta-analysis; FDA, US Food and Drug Administration; ICER, incremental cost-effectiveness ratio; OIR, only in research; QALY, quality-adjusted life-year; RR, relative risk; VOI, value of information.

### Sensitivity Analyses

The figures illustrating the results of the sensitivity analyses are provided in the Appendix.

- A **WTP threshold** of $150,000/QALY led to time point K9 now being considered cost-effective and additional prospective VOI time points suggesting approval (K11-K17+K19); however, retrospective VOI remained unchanged (Appendix Table 5).
- **Extended trial duration** resulted in more frequent approval, attributed to the few remaining future patients after the trial ends. For example, the number of future patients calculated in K11-K13, K15, and K19 was between only 1,000 and 3,000. When few patients benefited from future research, the costs of a new trial did not outweigh the benefits even when EVPPI was high. Loss, therefore, increased for the prospective VOI approach (Appendix Table 6).
- **Including only the trials listed by the FDA’s press release** showed that the FDA’s decision for EUA was inconsistent with both CMA and VOI or could be interpreted as late versus the CMA results if we extrapolate our analysis beyond our timeline until approval in December 2022. Prospective VOI did initially recommend EUA at the same time as CMA but did not recommend approval at any time point (Appendix Table 7).
- **Not extending the IHME predictions that ended mid-peak** (stop-and-drop assumption after the IHME predictions) did not alter the suggested optimal strategies but reduced the number of patients who could benefit at those time points and related values (Appendix Table 8).
- The traditional meta-analysis forest plot illustrates the **individual contribution of each study**. When examined in the model, the timeline plot now shows the results of the optimal suggested strategy if the recommendation of each approach was based on any of the individual studies (Appendix Table 9).
- The CMA was run **without using the Knapp-Hartung adjustment**, which increases the uncertainty intervals, especially when few studies have been included. This led to approval being suggested at earlier time points. As uncertainty was smaller, EVPPI was lower, and loss from choosing the suboptimal strategies at approval time points decreased (Appendix Table 10).
- Finally, in the subgroup analysis in which **only IL-6-(R-) inhibitors** tocilizumab, sarilumab, and siltuximab articles were eligible, 16 articles were included. The overall pooled result was more uncertain, resulting in a continued AWR recommendation in the CMA approach. Loss was largest in either the policy or CMA approach, depending on the time point (Appendix Table 11).

## Discussion

Policy makers face complex decisions under conditions of imperfect information. However, we can distinguish different approaches to aid their choices and maximize the chances of evidence-based decisions that consider the consequences of those decisions.

Our analysis demonstrated that FDA decisions were not always optimal, based on both reference standard hindsight knowledge and real-time VOI analysis. Our analysis demonstrates the consequences of delay in policy making: not making a decision should be considered an active decision to maintain the status quo. This means that when the FDA takes longer to provide EUA than VOI approaches, an active decision is taken to continue using MAbs only in research settings, foregoing the potential benefits to the population when treatment seems promising.

Guiding decision making based on CMA alone would have led to the largest opportunity loss of all strategies. Had we used VOI analysis prospectively, suboptimal decisions would still have been made due to imperfect information, but the expected loss in value would have been substantially reduced. The reference standard, retrospective VOI analysis, demonstrated that either OIR or AWR was the optimal strategy depending on the point in time.

The difference in recommendations between the retrospective and prospective VOI is driven by the number of anticipated current (those admitted to the hospital while the new trial is ongoing) and future patients (those who would benefit from the new trial’s results). Therefore, the discrepancy between these 2 approaches (K15-K17), in which the prospective VOI suggests approval and retrospective VOI suggests AWR, stems from hospitalization forecasts. This underscores the importance of accurate forecasts when considering the value of future research or at least the acknowledgment of the uncertainty in our predictions. Even when a previous time point may suggest approval, it is vital to reassess if there are changes in other parameters such as the estimated number of patients. The need to revisit and update the analysis becomes especially relevant when dealing with rapidly changing forecasts during a pandemic. Had we relied solely on CMA, our decision to approve without further research would have been independent of any forecast; thus, we may not have found new reasons to revisit our decision, and approval might not have been reversed. According to the reference standard, the final choice at the end of our timeline was to withdraw EUA.

### Strengths

To the best of our understanding, this is the first time these 3 real-time approaches—policy, CMA, and prospective VOI—were applied together across a timeline of evidence accrual along with a fourth, reference standard retrospective VOI approach. Our novel comparison to a reference standard helps us better understand the added value of VOI methods in decision-making processes. Implementing these methods as extensions to meta-analyses can facilitate a deeper understanding of the consequences of historical decisions and provide valuable insights for future decision-making methodologies, within a pandemic scenario and beyond.

The integration of CEA and VOI in treatment approval and future research decisions presents an important advantage over the reliance solely on CMA and statistical significance considerations.^5,59^

These techniques provide a more comprehensive evaluation by factoring in costs, the probability of treatment being cost-effective, additional uncertainties surrounding other parameters, and the consequences of each potential decision.^5,59^

By using CMA rather than traditional meta-analysis, we were able to show evidence accrual over time while considering the full body of available evidence. Notably, our exhaustive CMA did not exhibit publication bias. This contrasts with our sensitivity analysis that includes only the 4 trials mentioned in the FDA’s press release that announces EUA for tocilizumab.

### Limitations

In addition to the limitations underlying the decision-analytic model and VOI highlighted in Dijk et al.,^3^ this study brings its own set of methodological and practical limitations to consider.

#### Methodological limitations

Our study is a methodological showcase highlighting the choice of strategy in a timeline in the past, which limits its direct practical relevance. We have simplified the analysis where we deemed appropriate for a methodological article. We focused VOI on the treatment effect measure that would be investigated with future RCTs, but other parameters could also be chosen as targets to reduce uncertainty.

Our meta-analysis included some articles that made multiple treatment comparisons, such as dosage A versus usual care and dosage B versus usual care. By incorporating these 2 comparisons in our meta-analysis, we have double counted the number of controls, artificially increasing our sample size.

Our article calculates EVs of new decisions made immediately when new evidence becomes available. However, decision making and implementation take time and resources. Our analysis does not consider the cost to conduct CMA or VOI analyses themselves. Although our conclusion states that the policy approach did not make the appropriate decisions and delayed EUA, we do not consider the time frame it would take to act on VOI recommendations or the time required to develop a working decision analytic model. The first COVID-19 VOI model preprint became available only March 2022, beyond the scope of this timeline.^3^ By providing our full code, collected data, and this framework, we hope that analyses performed in future crisis situations can build on this existing work and enable more rapid decision making.

Given the distinct nature of each method, the comparisons are influenced by the inherent limitations of each approach.

This article provides evidence that prospective VOI provides additional value above more traditional approaches to decision making. Yet we also acknowledge that the prospective VOI was the most similar of all 3 approaches to what we set to be the reference standard. The fact that this approach considers prospective VOI to be the most effective strategy could be criticized as a self-fulfilling prophecy. Our choice of reference standard may not be universally applicable or agreed upon, which affects the interpretation of our results.

Lastly, the methods discussed in this article form a simplification of complex decisions. Our VOI assumes that decisions are based mainly on expected monetary and health outcomes but ignores other potential factors such as political, social, or resource-constraining considerations.

#### Limitations for practical implications and generalizability

While this article is presented as a methodological article, there are important assumptions and limitations that should be noted before considering any implications to practice.

The parameters in our analysis that change over time are treatment effectiveness (CMA) and predicted number of patients. We deliberately chose to vary only these parameters to underscore the differences between the approaches. However, other parameters that may change over time such as utility estimates, patient demographics, vaccination status, circulating SARS-CoV-2 variants, and changing usual care can also influence the analysis. Neither our CMA nor VOI explicitly captures all time-varying treatment effects due to evolving treatment protocols or patient characteristics. New developments and updates to the analysis with alternative scenarios remain important when new information arises. While VOI offers an advantage in adjusting model inputs to explore their impact on the suggested optimal strategy, it is crucial to acknowledge that both VOI and meta-analysis primarily focus on reducing statistical uncertainty and are less adept at detecting sources of bias or predicting unforeseen circumstances.

MAbs specifically are at even more risk than other treatments in the COVID-19 armamentarium for changing effectiveness over time. New variants of the SARS-CoV-2 virus can emerge due to mutations in the virus’s genome.^10^ An important practical limitation of our study is that our timeline ends on January 1, 2022. The FDA has since approved tocilizumab for COVID-19 and provided EUA to some other MAbs mainly for nonhospitalized patients but also revoked authorizations of MAbs such as sotrovimab and bebtelovimab. An important reason for discontinuation of EUA was when new variants, for example, Omicron subvariants BQ.1 and BQ1.1, showed a large reduction in (expected) susceptibility.^60^

Our analysis in a pandemic setting differs in several aspects from that in a nonpandemic setting while also sharing some commonalities. While we saw successful examples of multicenter trials such as RECOVERY,^39,61^ the pandemic also introduced a surge in numerous, small, underpowered trials, creating a unique landscape. Nonpandemic settings may allow for more efficient coordination of large, multicenter RCTs. In the pandemic, the highest number of patients was admitted to the hospital in the short term (during the new trial), whereas in general settings, more patients are expected in the future, even if the trial results will likely take longer to publish their results. These differences will influence the EV of early implementation and the collection of further evidence. A better stability in the predicted number of patients in the future will likely also reduce the fluctuation between optimal strategies in prospective VOI in nonpandemic settings. While we anticipate partial generalizability of our findings to nonpandemic settings, applications in other settings are needed to support these expectations.

We used a health care perspective with mainly US-based parameters. Although our modeling approach can be applied in different settings, our results cannot immediately be applied to different countries or globally, as for example, the costs of care, forecasts, and WTP thresholds will likely differ.

Our model identifies optimal strategies for research and treatment on the assumption that decision making and consequence bearing are centralized in the same entity. However, in reality this is not always the case. Specifically, an agency such as the FDA is not necessarily the entity deciding on research funding or the entity deciding on the incorporation of newly approved drugs in treatment guidelines. The decision-making landscape is in fact a complex network of various organizations, each with its own agenda and cost-outcome considerations. Our model may therefore not fully capture the diversity and nuance of the multistakeholder context within the health care system.

In our meta-analysis, we pooled results from all articles investigating MAbs eligible for inclusion, whereas from a pharmacologic standpoint, various types of MAbs can be distinguished along the pathways on which they work. Thus, we assumed sufficient homogeneity across these studies to allow for pooling of the results but used a random effects model to account for heterogeneity and performed sensitivity analysis to evaluate the effect of our assumption. Our search did not identify MAbs belonging to the neutralizing MAbs, likely because they are applied in outpatient settings. We performed a sensitivity analysis for the second major group, immune modulators, working through the IL-6-(receptor-)inhibition. In this analysis, the final CMA recommendation was AWR instead of approval due to remaining statistical uncertainty.

While treatment effectiveness is based on several different MAbs, we adopt the other parameters such as treatment cost and hospital stay in the decision-analytic model based on the most frequently cited treatment tocilizumab. In addition, the policy decision also applies to the EUA and approval of tocilizumab; however, in reality, each MAb treatment requires its own approval process in treating COVID-19, even if prior off-label use was possible.

Our analysis uses a trial duration of 2 months in our VOI, which can be subject to debate. This modeling choice had practical roots in the fact that prospective IHME predictions often projected only 4 months into the future, and we felt that at least half of these predictions should consider future patients. It would technically be possible to collect 28-d mortality figures from a new trial; however, this would require rapid decisions and trial rollouts to accomplish, and we will likely underestimate the number of patients in our model. The need for rapid trial rollout and large sample sizes as suggested by the EVSI supports the call for globally optimal trial design using for example a platform design, which could increase the speed at which decisions are made, reduce fixed trial costs, and increase the strength and implementation of evidence with large definitive trials.^61^

In addition, by choosing mortality as a singular endpoint for further investigation in further RCTs, we neglect the value that could be gained in collecting further information about other relevant endpoints such as morbidity or length of hospital stay. Neither do we consider alternative study types than RCTs that could investigate consequences such as long-term quality of life or costs.

Finally, our analysis assumes that the costs associated with the implementation and reversal of new decisions are negligible, as they concern guidelines and policy changes rather than physical interventions. Nevertheless, this assumption might overly simplify the real-world scenario, as we neglected costs related to policy change execution and time lags between the decision-making processes, its implementation, and the observable effects of those changes. We did not consider the consequences for trials that had already been initiated, including early termination when new evidence became available.

### Future Directions

This article, along with the accompanying code and data, offers a promising framework for employing and comparing multiple evidence-assessment methodologies including CMA and VOI. Given that this article pioneers this approach, it necessitates further validation across diverse settings or by applying it to alternative models. Further developments that could enhance the practical implementation of this model include its integration with a foundational susceptible-exposed-infectious-recovered (SEIR) model, which would anticipate the count of future patients. This integration would enable rapid updates to the results when forecasts change. Our code and data are made available to this end.

## Conclusion

Our retrospective VOI analysis (reference standard) showed that across the 2020 to 2022 timeline, either OIR or AWR was the strategy that yielded the highest expected net benefit among potential combined approval-research strategies. The discrepancy between the suggested strategy by the policy and retrospective VOI approaches indicates that resources were used suboptimally. Using only CMA to inform decisions on treatment research-approval would have led to the largest opportunity loss of all strategies, followed by the current FDA policy approach. The use of (prospective) VOI in future research-approval decisions should be considered as an extension of meta-analyses that also incorporates the consequences of each decision. Although suboptimal strategies would still have been suggested at certain time points in our MAb example, EV loss would have been reduced substantially.

## Supplemental Material

sj-pdf-1-mdm-10.1177_0272989X241255047 – Supplemental material for Making Drug Approval Decisions in the Face of Uncertainty: Cumulative Evidence versus Value of Information Supplemental material, sj-pdf-1-mdm-10.1177_0272989X241255047 for Making Drug Approval Decisions in the Face of Uncertainty: Cumulative Evidence versus Value of Information by Stijntje W. Dijk, Eline Krijkamp, Natalia Kunst, Jeremy A. Labrecque, Cary P. Gross, Aradhana Pandit, Chia-Ping Lu, Loes E. Visser, John B. Wong and M. G. Myriam Hunink in Medical Decision Making

sj-xlsx-2-mdm-10.1177_0272989X241255047 – Supplemental material for Making Drug Approval Decisions in the Face of Uncertainty: Cumulative Evidence versus Value of Information Supplemental material, sj-xlsx-2-mdm-10.1177_0272989X241255047 for Making Drug Approval Decisions in the Face of Uncertainty: Cumulative Evidence versus Value of Information by Stijntje W. Dijk, Eline Krijkamp, Natalia Kunst, Jeremy A. Labrecque, Cary P. Gross, Aradhana Pandit, Chia-Ping Lu, Loes E. Visser, John B. Wong and M. G. Myriam Hunink in Medical Decision Making
