# Supplementary material for: Making Drug Approval Decisions in the Face of Uncertainty: Cumulative Evidence versus Value of Information
Source: Med Decis Making. 2024 Jun 3;44(5):512–28. doi: 10.1177/0272989X241255047 (PMC11283736; doi:10.1177/0272989X241255047)
Supplement: sj-pdf-1-mdm-10.1177_0272989X241255047 – Supplemental material for Making Drug Approval Decisions in the Face of Uncertainty: Cumulative Evidence versus Value of Information [file sj-pdf-1-mdm-10.1177_0272989X241255047.pdf]

Appendix

Meta-analysis **2**

Appendix, Table 1: Search Records Identified 2

Appendix, Table 2: Search Syntax 2

Appendix, Figure 1: PRISMA Flowchart. 5

Appendix, Table 3: Study Characteristics 6

Appendix, Figure 2: Global distribution of included studies. 7

Appendix, Table 4: Risk of Bias 8

Appendix, Figure 3: Funnel plot for demonstrating publication bias. 9

Appendix, Figure 4: Traditional (non-cumulative) meta-analysis Forest plot. 10

Appendix, Figure 5: Subset FDA trials 11

Appendix, Figure 6: Meta-regression of Severity. 12

Decision-analytic model **13**

Appendix, Figure 7: State-transition diagram. 13

Appendix, Figure 8: IHME predictions – Retrospective. 14

Appendix, Figure 9: IHME predictions – Prospective. 15

Appendix, Figure 10: Extended and non-extended peak IHME predictions. 16

Appendix, Figure 11: Incremental Cost-effectiveness planes (Rx vs Cx) for all timepoints using cumulative meta-analysis results. 17

Appendix, Figure 12: Incremental Cost-effectiveness planes (Rx vs Cx) for all timepoints using cumulative meta-analysis results with uncertainty ellipse. 18

Appendix, Figure 13: Cost-Effectiveness Curves and Frontiers 19

Appendix, Figure 14. EVPSI plots (QALY) over time 20

Appendix, Figure 15: Prospective analysis, full results table. 21

Appendix, Figure 16: Retrospective analysis, full results table. 21

Sensitivity analyses **22**

Appendix, Table 5: Sensitivity Analysis – WTP $150.000. 22

Appendix, Table 6: Sensitivity Analysis – 3-month trial duration 23

Appendix, Table 7: Sensitivity Analysis – FDA included trials only 24

Appendix, Table 8: Sensitivity Analysis – No extended peaks (unadjusted IHME data) 25

Appendix, Table 9: Sensitivity Analysis – Traditional meta-analysis (non-cumulative) 26

Appendix, Table 10: Sensitivity Analysis – Basic CMA (no Knapp Hartung adjustment) 27

Appendix, Table 11: Sensitivity Analysis – IL-6-(R-)inhibitors 28

Reporting guidelines **29**

Appendix, Table 12: PRISMA checklist 29

Appendix, Table 13: CHEERS and CHEERS VOI statement 32

Meta-analysis

Appendix, Table 1: Search Records Identified

Database searched	via	Years of coverage	Records	Records after duplicates removed
Embase	Embase.com	1971 - Present	3365	3303
Medline ALL	Ovid	1946 - Present	1728	334
Web of Science Core Collection*	Web of Knowledge	1975 - Present	1506	456
Cochrane Central Register of Controlled Trials	Wiley	1992 - Present	1623	1114
WHO Covid-19 database	search.bvsalud.org	2008 - Present	1911	864
Other sources: Google Scholar (500 top-ranked)			500	432
**Total**			**10633**	**6503**

*Science Citation Index Expanded (1975-present) ; Social Sciences Citation Index (1975-present) ; Arts & Humanities Citation Index (1975-present) ; Conference Proceedings Citation Index- Science (1990-present) ; Conference Proceedings Citation Index- Social Science & Humanities (1990-present) ; Emerging Sources Citation Index (2015-present)

Appendix, Table 2: Search Syntax

**Embase 3365**	('severe acute respiratory syndrome coronavirus 2'/de OR '2019 novel coronavirus'/de OR 'covid 19'/de OR 'coronavirus disease 2019'/exp OR 'SARS-CoV-2 vaccine'/exp OR 'SARS-CoV-2 antibody'/de OR 'SARS coronavirus 2 test kit'/de OR 'anti-SARS-CoV-2 agent'/de OR (COVID-19* OR COVID19* OR 2019-ncov* OR 2019ncov* OR 2019-novel-coronavirus* OR 2019-novel-corona-virus* OR (wuhan* NEAR/3 (coronavirus* OR corona-virus*)) OR (Wuhan* NEAR/3 seafood NEAR/3 pneumonia) OR Coronavirus-Disease-2019 OR Corona-virus-Disease-2019 OR Coronavirus-Disease-19 OR Corona-virus-Disease-19 OR SARS-CoV-2 OR SARS-CoV2 OR SARS-Coronavirus-2 OR sars2 OR sars-2 OR ((new OR novel) NEAR/3 (coronavirus OR corona-virus*) NEAR/3 pneumonia) OR severe-acute-respiratory-syndrome-cov*-2 OR severe-acute-respiratory-syndrome-cov2):ab,ti,kw OR (('Coronaviridae'/exp OR 'Coronavirus infection'/exp OR (coronavir*):ab,ti,kw) AND [2020-2030]/py)) AND ('hospital patient'/exp OR 'intensive care unit'/exp OR 'intensive care'/exp OR 'critical care'/exp OR 'artificial ventilation'/exp OR 'critically ill patient'/exp OR 'hospitalization'/exp OR 'hospital admission'/exp OR 'in-hospital mortality'/exp OR (hospitaliz* OR hospitalis* OR hospital-patient* OR inpatient* OR intensive-care* OR ICU OR critical-care* OR ((mechanical* OR artificial*) NEAR/3 (ventilation* OR ventilated)) OR ((critical* or severely) NEAR/3 (ill)) OR hospital-stay* OR hospital-admission* OR hospital-admit*):ab,ti,kw) AND ('drug therapy'/exp OR 'coronavirus disease 2019'/'drug therapy' OR 'anticoronavirus agent'/exp OR 'drug'/exp OR 'antivirus agent'/exp OR 'dipeptidyl carboxypeptidase inhibitor'/exp OR 'anakinra'/de OR 'azithromycin'/de OR 'colchicine'/de OR 'corticosteroid'/exp OR 'doxycycline'/de OR 'ivermectin'/de OR 'hydroxychloroquine'/de OR 'azithromycin'/de OR 'cytokine receptor antagonist'/exp OR 'Janus kinase inhibitor'/exp OR 'nitazoxanide'/de OR 'sulodexide'/de OR 'ascorbic acid'/exp OR 'vitamin D'/exp OR 'convalescent plasma'/exp OR 'convalescent plasma therapy'/exp OR 'anticoagulant agent'/exp OR 'anticoagulants therapeutic use'/exp OR 'monoclonal antibody'/exp OR (drug OR drugs* OR medication* OR medicinal OR ((pharmaco* OR pharmaceut* OR drug) NEAR/3 (therap* OR treatment* OR dose* OR intervent*)) OR pharmacotherap* OR pharmacotreatment* OR ((anti-covid* OR anticovid* OR antivirus* OR antiviral* OR anti-virus* OR anti-viral*) NEAR/3 (therap* OR molecul* OR agent* OR compound* OR ingredient*)) OR viral-inhibit* OR antivirals OR virustatic* OR virucide* OR ACE-inhibitor* OR angiotensin-converting-enzyme-inhibit* OR dipeptidyl-carboxypeptidase-inhibit* OR anakinra* OR azithromycin* OR colchicine* OR corticosteroid* OR doxycycline* OR ivermectin* OR favipiravir* OR hydroxychloroquine* OR azithromycin* OR cytokine-receptor-antagonist* OR cytokine-antagonist* OR interleukin-receptor-antagonist* OR IL-6i* OR Tocilizumab* OR beta1a-interferon* OR interferon-beta* OR JAK-inhibit* OR Janus-kinase-inhibit* OR Lopinavir* OR Ritonavir* OR nitazoxanide* OR proxalutimide* OR rhG-CSF* OR sulodexide* OR ascorbic-acid* OR vitamin-C* OR vitamin-D* OR baricitinib* OR Dexamethasone* OR Remdesivir* OR convalescent-plasma* OR anticoagulant* OR anti-coagulant* OR monoclonal-antibod*):ab,ti,kw) AND ('randomized controlled trial'/exp OR 'Controlled clinical trial'/exp OR 'Crossover procedure'/de OR 'Double-blind procedure'/de OR 'Single-blind procedure'/de OR 'systematic review'/de OR 'meta analysis'/exp OR (RCT OR RCTs OR random* OR factorial* OR crossover* OR (cross NEXT/1 over*) OR placebo* OR ((doubl* OR singl*) NEXT/1 blind*) OR assign* OR allocat* OR volunteer* OR trial OR groups):ab,ti,kw OR (systematic-review* OR meta-analy*):ti) NOT ((animal/exp OR animal*:de OR nonhuman/de) NOT ('human'/exp)) NOT (('juvenile'/exp OR (juvenil* OR adolescen* OR preadolescen* OR youth* OR

	child* OR schoolchild* OR minors OR teen OR teens OR teenager* OR infan* OR toddler* OR pediatr* OR paediatr* OR puber* OR baby OR babies OR girl* OR boy* OR newborn* OR neonate* OR premature* OR pre-matur* OR kid OR kids OR underag* OR kindergar* OR pubescen* OR prepubesc* OR school* OR preschool* OR highschool* OR suckling OR PICU OR NICU OR PICUs OR NICUs):ab,ti,kw) NOT ('adult'/exp OR (adult* OR elderl* OR man OR men OR woman OR women):ab,ti,kw))
**Medline 1728**	(exp SARS-CoV-2/ OR exp COVID-19/ OR (COVID-19* OR COVID19* OR 2019-ncov* OR 2019ncov* OR 2019-novel-coronavirus* OR 2019-novel-corona-virus* OR (wuhan* ADJ3 (coronavirus* OR corona-virus*)) OR (Wuhan* ADJ3 seafood ADJ3 pneumonia) OR Coronavirus-Disease-2019 OR Corona-virus-Disease-2019 OR Coronavirus-Disease-19 OR Corona-virus-Disease-19 OR SARS-CoV-2 OR SARS-CoV2 OR SARS-Coronavirus-2 OR sars2 OR sars-2 OR ((new OR novel) ADJ3 (coronavirus OR corona-virus*) ADJ3 pneumonia) OR severe-acute-respiratory-syndrome-cov*-2 OR severe-acute-respiratory-syndrome-cov2).ab,ti,kf.) AND (Inpatients/ OR exp Intensive Care Units/ OR Critical Care/ OR Critical Care Nursing/ OR exp Respiration, Artificial/ OR Critical Illness/ OR exp Hospitalization/ OR Hospital Mortality/ OR (hospitaliz* OR hospitalis* OR hospital-patient* OR inpatient* OR intensive-care* OR ICU OR critical-care* OR ((mechanical* OR artificial*) ADJ3 (ventilation* OR ventilated)) OR ((critical* or severely) ADJ3 (ill)) OR hospital-stay* OR hospital-admission* OR hospital-admit*).ab,ti,kf.) AND (exp Drug Therapy/ OR exp COVID-19/dt OR exp SARS-CoV-2/de OR exp Pharmaceutical Preparations/ OR exp Antiviral Agents/ OR exp Angiotensin-Converting Enzyme Inhibitors/ OR Interleukin 1 Receptor Antagonist Protein/ OR Azithromycin/ OR exp Colchicine/ OR exp Adrenal Cortex Hormones/ OR Doxycycline/ OR Ivermectin/ OR Hydroxychloroquine/ OR Azithromycin/ OR Janus Kinase Inhibitors/ OR nitazoxanide.nm OR glucuronyl glucosamine glycan sulfate.nm OR exp Ascorbic Acid/ OR exp Vitamin D/ OR COVID-19 serotherapy.ps OR exp Anticoagulants/ OR exp Antibodies, Monoclonal/ OR (drug OR drugs* OR medication* OR medicinal OR ((pharmaco* OR pharmaceut* OR drug) ADJ3 (therap* OR treatment* OR dose* OR intervent*)) OR pharmacotherap* OR pharmacotreatment* OR ((anti-covid* OR anticovid* OR antivirus* OR antiviral* OR anti-virus* OR anti-viral*) ADJ3 (therap* OR molecul* OR agent* OR compound* OR ingredient*)) OR viral-inhibit* OR antivirals OR virustatic* OR virucide* OR ACE-inhibitor* OR angiotensin-converting-enzyme-inhibit* OR dipeptidyl-carboxypeptidase-inhibit* OR anakinra* OR azithromycin* OR colchicine* OR corticosteroid* OR doxycycline* OR ivermectin* OR favipiravir* OR hydroxychloroquine* OR azithromycin* OR cytokine-receptor-antagonist* OR cytokine-antagonist* OR interleukin-receptor-antagonist* OR IL-6i* OR Tocilizumab* OR beta1a-interferon* OR interferon-beta* OR JAK-inhibit* OR Janus-kinase-inhibit* OR Lopinavir* OR Ritonavir* OR nitazoxanide* OR proxalutimide* OR rhG-CSF* OR sulodexide* OR ascorbic-acid* OR vitamin-C* OR vitamin-D* OR baricitinib* OR Dexamethasone* OR Remdesivir* OR convalescent-plasma* OR anticoagulant* OR anti-coagulant* OR monoclonal-antibod*).ab,ti,kf.) AND (exp Randomized Controlled Trial/ OR exp Controlled Clinical Trial/ OR Cross-Over Studies/ OR Double-Blind Method/ OR Single-Blind Method/ OR Systematic Review/ OR Meta-Analysis/ OR (RCT OR RCTs OR random* OR factorial* OR crossover* OR (cross ADJ over*) OR placebo* OR ((doubl* OR singl*) ADJ blind*) OR assign* OR allocat* OR volunteer* OR trial OR groups).ab,ti,kf. OR (systematic-review* OR meta-analy*).ti.) NOT (exp animals/ NOT humans/) NOT ((exp Infant/ OR exp Child/ OR exp Adolescent/ OR (juvenil* OR adolescen* OR preadolescen* OR youth* OR child* OR schoolchild* OR minors OR teen OR teens OR teenager* OR infan* OR toddler* OR pediatr* OR paediatr* OR puber* OR baby OR babies OR girl* OR boy* OR newborn* OR neonate* OR premature* OR pre-matur* OR kid OR kids OR underag* OR kindergar* OR pubescen* OR prepubesc* OR school* OR preschool* OR highschool* OR suckling OR PICU OR NICU OR PICUs OR NICUs).ab,ti,kf.) NOT (exp Adult/ OR (adult* OR elderl* OR man OR men OR woman OR women).ab,ti,kf.))
**Cochrane 1623**	((COVID NEXT 19* OR COVID19* OR 2019 NEXT ncov* OR 2019ncov* OR 2019 NEXT novel NEXT coronavirus* OR 2019 NEXT novel NEXT corona NEXT virus* OR (wuhan* NEAR/3 (coronavirus* OR corona NEXT virus*)) OR (Wuhan* NEAR/3 seafood NEAR/3 pneumonia) OR Coronavirus NEXT Disease NEXT 2019 OR Corona NEXT virus NEXT Disease NEXT 2019 OR Coronavirus NEXT Disease NEXT 19 OR Corona NEXT virus NEXT Disease NEXT 19 OR SARS NEXT CoV NEXT 2 OR SARS NEXT CoV2 OR SARS NEXT Coronavirus NEXT 2 OR sars2 OR sars NEXT 2 OR ((new OR novel) NEAR/3 (coronavirus OR corona NEXT virus*) NEAR/3 pneumonia) OR severe NEXT acute NEXT respiratory NEXT syndrome NEXT cov* NEXT 2 OR severe NEXT acute NEXT respiratory NEXT syndrome NEXT cov2):ab,ti,kw) AND ((hospitaliz* OR hospitalis* OR hospital NEXT patient* OR inpatient* OR intensive NEXT care* OR ICU OR critical NEXT care* OR ((mechanical* OR artificial*) NEAR/3 (ventilation* OR ventilated)) OR ((critical* or severely) NEAR/3 (ill)) OR hospital NEXT stay* OR hospital NEXT admission* OR hospital NEXT admit*):ab,ti,kw) AND ((drug OR drugs* OR medication* OR medicinal OR ((pharmaco* OR pharmaceut* OR drug) NEAR/3 (therap* OR treatment* OR dose* OR intervent*)) OR pharmacotherap* OR pharmacotreatment* OR ((anti NEXT covid* OR anticovid* OR antivirus* OR antiviral* OR anti NEXT virus* OR anti NEXT viral*) NEAR/3 (therap* OR molecul* OR agent* OR compound* OR ingredient*)) OR viral NEXT inhibit* OR antivirals OR virustatic* OR virucide* OR ACE NEXT inhibitor* OR angiotensin NEXT converting NEXT enzyme NEXT inhibit* OR dipeptidyl NEXT carboxypeptidase NEXT inhibit* OR anakinra* OR azithromycin* OR colchicine* OR corticosteroid* OR doxycycline* OR ivermectin* OR favipiravir* OR hydroxychloroquine* OR azithromycin* OR cytokine NEXT receptor NEXT antagonist* OR cytokine NEXT antagonist* OR interleukin NEXT receptor NEXT antagonist* OR IL NEXT 6i* OR Tocilizumab* OR beta1a NEXT interferon* OR interferon NEXT beta* OR JAK NEXT inhibit* OR Janus NEXT kinase NEXT inhibit* OR Lopinavir* OR Ritonavir* OR nitazoxanide* OR proxalutimide* OR rhG NEXT CSF* OR sulodexide* OR ascorbic NEXT acid* OR vitamin NEXT C OR vitamin NEXT D OR baricitinib* OR Dexamethasone* OR Remdesivir* OR convalescent NEXT plasma* OR anticoagulant* OR anti NEXT coagulant* OR monoclonal NEXT antibod*):ab,ti,kw) NOT (((juvenil* OR adolescen* OR preadolescen* OR youth* OR child* OR schoolchild* OR minors OR teen OR teens OR teenager* OR infan* OR toddler* OR pediatr* OR paediatr* OR puber* OR baby OR babies OR girl* OR boy* OR newborn* OR neonate*

	OR premature* OR pre NEXT matur* OR kid OR kids OR underag* OR kindergar* OR pubescen* OR prepubesc* OR school* OR preschool* OR highschool* OR suckling OR PICU OR NICU OR PICUs OR NICUs):ab,ti,kw) NOT ((adult* OR elderl* OR man OR men OR woman OR women):ab,ti,kw))
**Web of Science 1506**	TS=(((COVID-19* OR COVID19* OR 2019-ncov* OR 2019ncov* OR 2019-novel-coronavirus* OR 2019-novel-corona-virus* OR (wuhan* NEAR/2 (coronavirus* OR corona-virus*)) OR (Wuhan* NEAR/2 seafood NEAR/2 pneumonia) OR Coronavirus-Disease-2019 OR Corona-virus-Disease-2019 OR Coronavirus-Disease-19 OR Corona-virus-Disease-19 OR SARS-CoV-2 OR SARS-CoV2 OR SARS-Coronavirus-2 OR sars2 OR sars-2 OR ((new OR novel) NEAR/2 (coronavirus OR corona-virus*) NEAR/2 pneumonia) OR severe-acute-respiratory-syndrome-cov*-2 OR severe-acute-respiratory-syndrome-cov2)) AND ((hospitaliz* OR hospitalis* OR hospital-patient* OR inpatient* OR intensive-care* OR ICU OR critical-care* OR ((mechanical* OR artificial*) NEAR/2 (ventilation* OR ventilated)) OR ((critical* or severely) NEAR/2 (ill)) OR hospital-stay* OR hospital-admission* OR hospital-admit*)) AND ((drug OR drugs* OR medication* OR medicinal OR ((pharmaco* OR pharmaceut* OR drug) NEAR/2 (therap* OR treatment* OR dose* OR intervent*)) OR pharmacotherap* OR pharmacotreatment* OR ((anti-covid* OR anticovid* OR antivirus* OR antiviral* OR anti-virus* OR anti-viral*) NEAR/2 (therap* OR molecul* OR agent* OR compound* OR ingredient*)) OR viral-inhibit* OR antivirals OR virustatic* OR virucide* OR ACE-inhibitor* OR angiotensin-converting-enzyme-inhibit* OR dipeptidyl-carboxypeptidase-inhibit* OR anakinra* OR azithromycin* OR colchicine* OR corticosteroid* OR doxycycline* OR ivermectin* OR favipiravir* OR hydroxychloroquine* OR azithromycin* OR cytokine-receptor-antagonist* OR cytokine-antagonist* OR interleukin-receptor-antagonist* OR IL-6i* OR Tocilizumab* OR beta1a-interferon* OR interferon-beta* OR JAK-inhibit* OR Janus-kinase-inhibit* OR Lopinavir* OR Ritonavir* OR nitazoxanide* OR proxalutimide* OR rhG-CSF* OR sulodexide* OR ascorbic-acid* OR vitamin-C* OR vitamin-D* OR baricitinib* OR Dexamethasone* OR Remdesivir* OR convalescent-plasma* OR anticoagulant* OR anti-coagulant* OR monoclonal-antibod*)) AND ((RCT OR RCTs OR random* OR factorial* OR crossover* OR (cross NEAR/1 over*) OR placebo* OR ((doubl* OR singl*) NEAR/1 blind*) OR assign* OR allocat* OR volunteer* OR trial OR groups)) NOT ((juvenil* OR adolescen* OR preadolescen* OR youth* OR child* OR schoolchild* OR minors OR teen OR teens OR teenager* OR infan* OR toddler* OR pediatr* OR paediatr* OR puber* OR baby OR babies OR girl* OR boy* OR newborn* OR neonate* OR premature* OR pre-matur* OR kid OR kids OR underag* OR kindergar* OR pubescen* OR prepubesc* OR school* OR preschool* OR highschool* OR suckling OR PICU OR NICU OR PICUs OR NICUs) NOT (adult* OR elderl* OR man OR men OR woman OR women)))
**Google Scholar**	COVID|COVID19|2019-ncov|2019ncov|coronavirus|SARS-CoV-2|SARS-CoV2<br>hospitalization|hospitalisation|inpatient|in-patient|intensive-care|ICU<br>drug|drugs|medication|medicinal|pharmaceutical|anticovid|antivirus|antiviral RCT|random|randomized|placebo|trial
**WHO-covid19 database: 1911**	https://search.bvsalud.org/global-literature-on-novel-coronavirus-2019-ncov/<br>(hospitaliz* OR hospitalis* OR inpatient* OR in-patient* OR intensive-care* OR ICU) AND (drug* OR medication* OR medicinal OR pharmaceutic* OR anticovid* OR antivirus* OR antiviral) AND (RCT* OR random* OR placebo* OR trial)

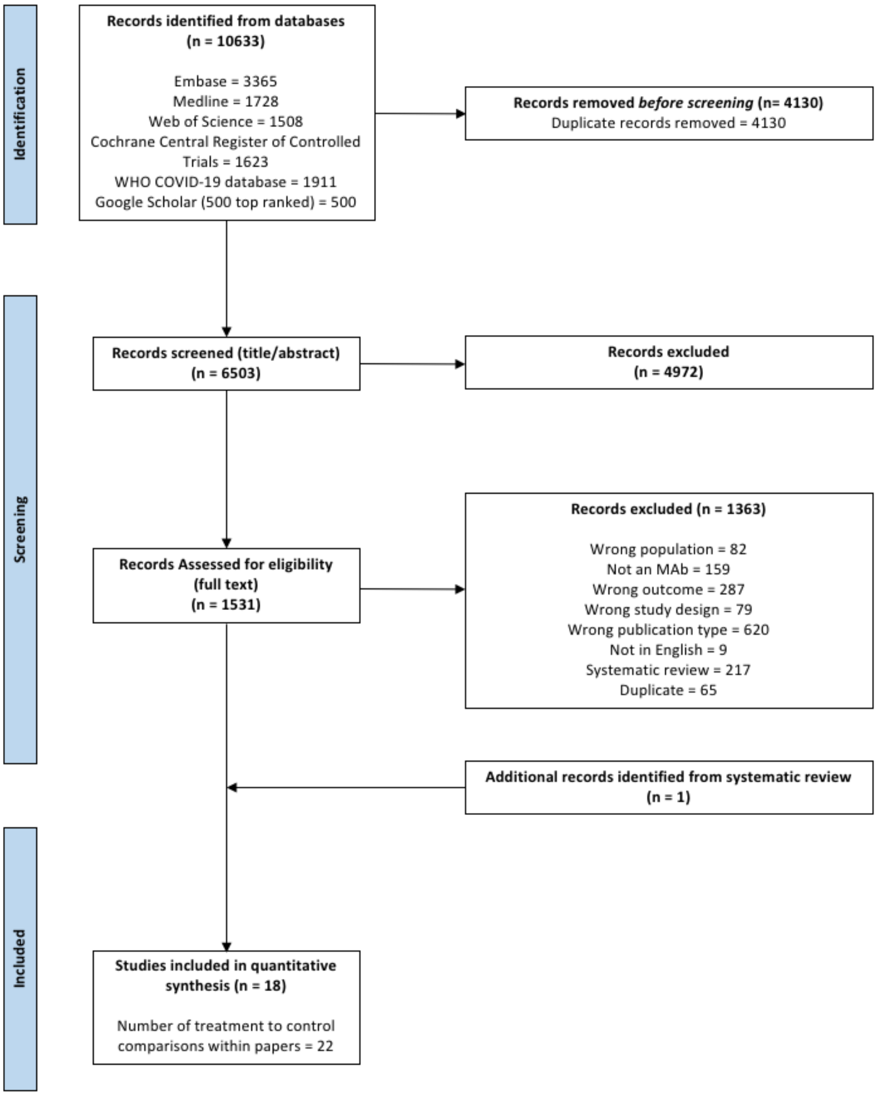

Appendix, Figure 1: PRISMA Flowchart.

The figure presents a flowchart with the results of the search and inclusion process. Note that 18 studies were included in the final analysis, which consisted of 22 treatment to control comparisons. MAb = Monoclonal Antibodies

Appendix, Table 3: Study Characteristics

k	Authors	Trial ID	Acronym	Location	Severity of disease at baseline	% severe patients	Start date	End date	Publication date	Total enrolled	Intervention arm	No. of subjects Intervention arm	Control arm	No. of subjects Control arm	Mortality Intervention arm	Mortality Control arm
1	Rosas et al.	NCT04320615	COVACTA	Canada, Denmark, France, Germany, Italy, Netherlands, Spain, UK, and USA	Severe disease based on SpO2 or PaO2/FiO2 levels	68.7	03-04-20	28-06-20	12-09-20	438	TCZ + CAU	294	Placebo + CAU	144	58	28
2	Vlaar et al.	NCT04333420	PANAMO	Netherlands	Severe disease based on PaO2/FiO2 level	60	31-03-20	24-04-20	28-09-20	30	Vilobelimab + CAU	15	CAU	15	2	4
3	Hermine et al.	NCT04331808	CORIMUNO TOCI 1	France	Moderate to severe based on WHO-CPS score without NIV or MV	0	31-03-20	18-04-20	20-10-20	131	TCZ + CAU	63	CAU	67	7	8
4	RCT-TCZ-COVID-19 Study Group	NCT04346355	RCT-TCZ	Italy	Moderate based on PaO2/FiO2 level and inflammatory markers, excluded ICU patients	0	31-03-20	11-06-20	20-10-20	126	TCZ	60	CAU	66	2	1
5	Salama et al.	NCT04372186	EMPACTA	USA, Brazil, Kenya, Mexico, Peru, South Africa	Moderate based on SpO2, excluded patients on CPAP, BiPAP or MV	26.5	14-05-20	18-08-20	23-10-20	377	TCZ + CAU	249	Placebo + CAU	128	26	11
6	Kumar et al.	CTRI/2020/05/024959	Kumar et al.	India	Moderate to severe based on PaO2/FiO2 levels and inflammatory markers	28.1	02-05-20	07-07-20	02-12-20	32	Itolizumab + BSC	20	CAU	10	0	3
7	REMAP CAP investigators Toci	NCT02735707	REMAP-CAP-TCZ	UK, Netherlands, Australia, New Zealand, Ireland, Saudi Arabia	Critically ill patients	100	09-03-20	19-11-20	09-01-21	755	TCZ	353	CAU	402	87	134
8	REMAP CAP investigators Sari	NCT02735707	REMAP-CAP-Sarilumab	UK, Netherlands, Australia, New Zealand, Ireland, Saudi Arabia	Critically ill patients	100	09-03-20	19-11-20	09-01-21	450	Sarilumab	48	CAU	402	11	134
9	Veiga et al.	NCT04403685	TOCIBRAS	Brazil	Severe or critical with evidence of pulmonary infiltrates + supplemental oxygen	48.1	08-05-20	17-07-20	20-01-21	129	TCZ + CAU	65	CAU	64	14	6
10	Recovery Collaborative Group	NCT04381936	RECOVERY	UK	Severe disease based on hypoxia and evidence of systemic inflammation	54.6	23-04-20	24-01-21	11-02-21	4116	TCZ + CAU	2022	CAU	2094	621	729
11	Soin et al.	CTRI/2020/05/025369	COVINTOC	India	Moderate to severe based on respiratory rate or SpO2, or presence of ARDS/septic shock	57	30-05-20	31-08-20	04-03-21	179	TCZ + CAU	91	CAU	88	11	15
12	Lescure et al. Sari400	NCT04327388	Lescure et al. Sari400	Argentina, Brazil, Canada, Chile, France, Germany, Israel, Italy, Japan, Russia, Spain	Severe group and critical group based on type of oxygen supplementation and need for ICU	25.3	28-03-20	03-07-20	04-03-21	257	Sarilumab 400mg	173	Placebo	84	14	7
13	Lescure et al. Sari200	NCT04327388	Lescure et al. Sari200	Argentina, Brazil, Canada, Chile, France, Germany, Israel, Italy, Japan, Russia, Spain	Severe group and critical group based on type of oxygen supplementation and need for ICU	26.7	28-03-20	03-07-20	04-03-21	243	Sarilumab 200mg	159	Placebo	84	16	7
14	Cremer et al.	NCT04399980; NCT04492514 ; NCT04463004	MASH-COVID	USA	Moderate to severe based on hypoxia and inflammatory markers	50	28-05-20	15-09-20	17-03-21	40	Mavrilimumab	21	Placebo	19	1	3
15	Patel et al.	NCT04376684	OSCAR	Argentina, Belgium, Brazil, Canada, Chile, France, India, Japan, Mexico, Netherlands, Peru, Poland, Russia, South Africa, Spain, UK, and USA	Moderate to severe disease based on hypoxemia and markers of systematic inflammation	100	28-05-20	13-01-21	17-04-21	793	Otilimab + SOC	395	Placebo + CAU	398	64	74
16	Temesgen et al.	NCT04351152	LIVE AIR	USA and Brazil	Moderate based on oxygen saturation	41	05-05-20	27-01-21	05-05-21	479	Lenzilumab + supportive care	236	Placebo + CAU	243	24	34
17	Boyapati et al. Sari400	NCT04315298	Boyapati et al. Sari400	USA	Severe disease	71.9	18-03-20	01-07-20	08-09-21	270	Sarilumab 400mg	180	Placebo	90	42	19
18	Boyapati et al. Sari200	NCT04315298	Boyapati et al. Sari200	USA	Severe disease	72.9	18-03-20	01-07-20	08-09-21	277	Sarilumab 200mg	187	Placebo	90	55	19
19	Lomakin et al.	NCT04397562	CORONA	Russia	Severe disease based on the presence of one of many criteria defined in the protocol	0	29-04-20	03-08-20	29-09-21	206	LVL + SOC	103	Placebo + CAU	103	4	4
20	Declereq et al. Toci	NCT04330638	COV-AID-TCZ	Belgium	Severe based on hypoxia & PaO2/FiO2 levels with signs of cytokine release syndrome	49.5	04-04-20	06-12-20	29-10-21	231	Tocilizumab (incl Anakinra) + CAU	113	Anakinra + CUA	118	13	14
21	Declereq et al. Siltux	NCT04330638	COV-AID-Siltux	Belgium	Severe based on hypoxia & PaO2/FiO2 levels with signs of cytokine release syndrome	49.5	04-04-20	06-12-20	29-10-21	229	Siltuximab (Incl Anakinra) + CAU	111	Anakinra + CUA	118	16	14
22	Stone et al.	NCT04356937	BACC BAY	USA	Moderate pneumonia, none ventilated at baseline	4.5	20-04-20	13-07-20	10-12-21	242	TCZ + CAU	161	Placebo + CAU	81	9	3

WHO-CPS score = World Health Organization COVID Progression Scale scoring, NIV = Non-invasive ventilation, MV = Mechanical ventilation, CPAP = Continuous positive airway pressure, BiPAP = Bilevel positive airway pressure, SpO2 = Oxygen saturation, PaO2/FiO2 = ratio of partial pressure of oxygen to fraction of inspired oxygen, ARDS = acute respiratory distress syndrome, CAU = Care as usual/usual care, Toci/TCZ = Tocilizumab, Sari = Sarilumab, LVL = Levilimab.

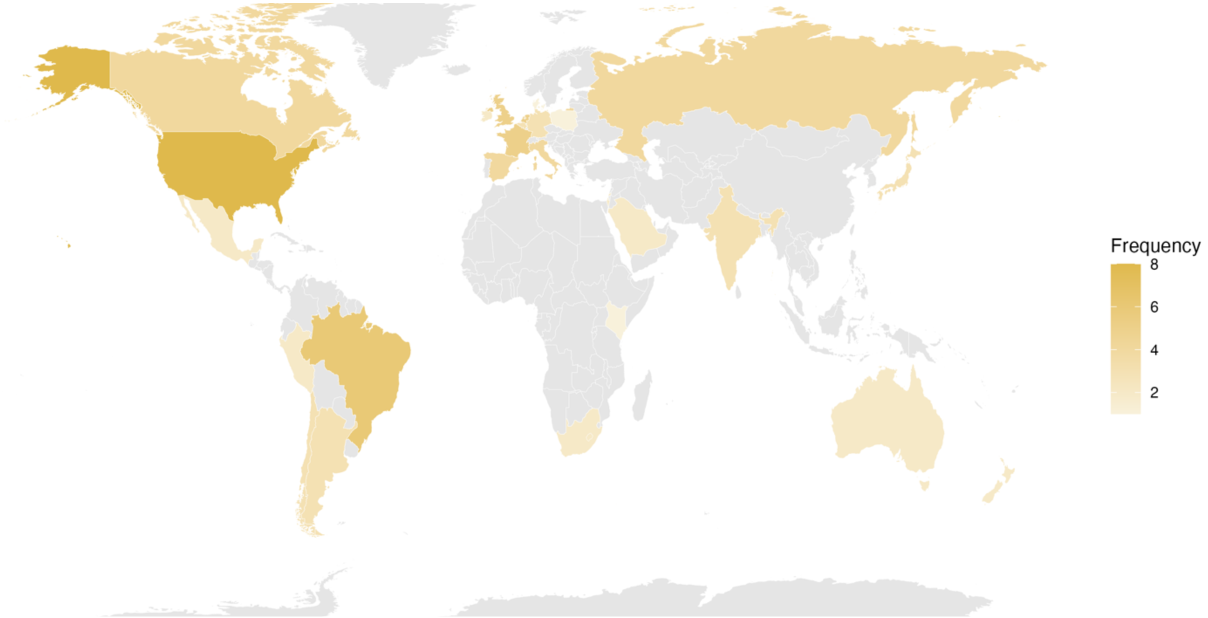

Appendix, Figure 2: Global distribution of included studies.

More frequently cited locations are represented by a darker shade. Non-cited countries are shown in grey.

Appendix, Table 4: Risk of Bias

Study No.	Authors	Domain 1	Domain 2	Domain 3	Domain 4	Domain 5	Overall Bias
1	Vlaar et al	Low	Low	Low	Low	Low	Low
2	Hermine et al	Low	Low	Low	Low	Low	Low
3	RCT-TCZ study group	Low	Some concerns	Low	Low	Low	Some concerns
4	Stone et al.	Low	Low	Low	Low	Low	Low
5	Salama et al.	Low	Low	Low	Low	Low	Low
6	Veiga et al.	Low	Low	Low	Low	Low	Low
7	REMAP-CAP investigators	Low	Low	Low	Low	Low	Low
8	Soin et al.	Low	Low	Low	Low	Low	Low
9	Lescure et al.	Low	Low	Low	Low	Low	Low
10	Kumar et al.	Low	Low	Low	Low	Low	Low
11	Cremer et al.	Low	Low	Low	Low	Low	Low
12	Patel et al.	Low	Low	Low	Low	Low	Low
13	Rosas et al.	Low	Low	Low	Low	Low	Low
14	RECOVERY group	Low	Low	Low	Low	Low	Low
15	Boyapati et al.	Low	Low	Low	Low	Low	Low
16	Lomakin et al.	Low	Some concerns	Low	Low	Low	Some concerns
17	Declercq et al.	Low	Low	Low	Low	Low	Low
18	Temesgen et al.	Low	Low	Low	Low	Low	Low

Risk of bias for each article. Domain 1 – Randomization process, Domain 2 – Deviations from intended interventions, Domain 3 – Missing outcome data, Domain 4 – Measurement of the outcome, Domain 5 – Selection of the reported result.

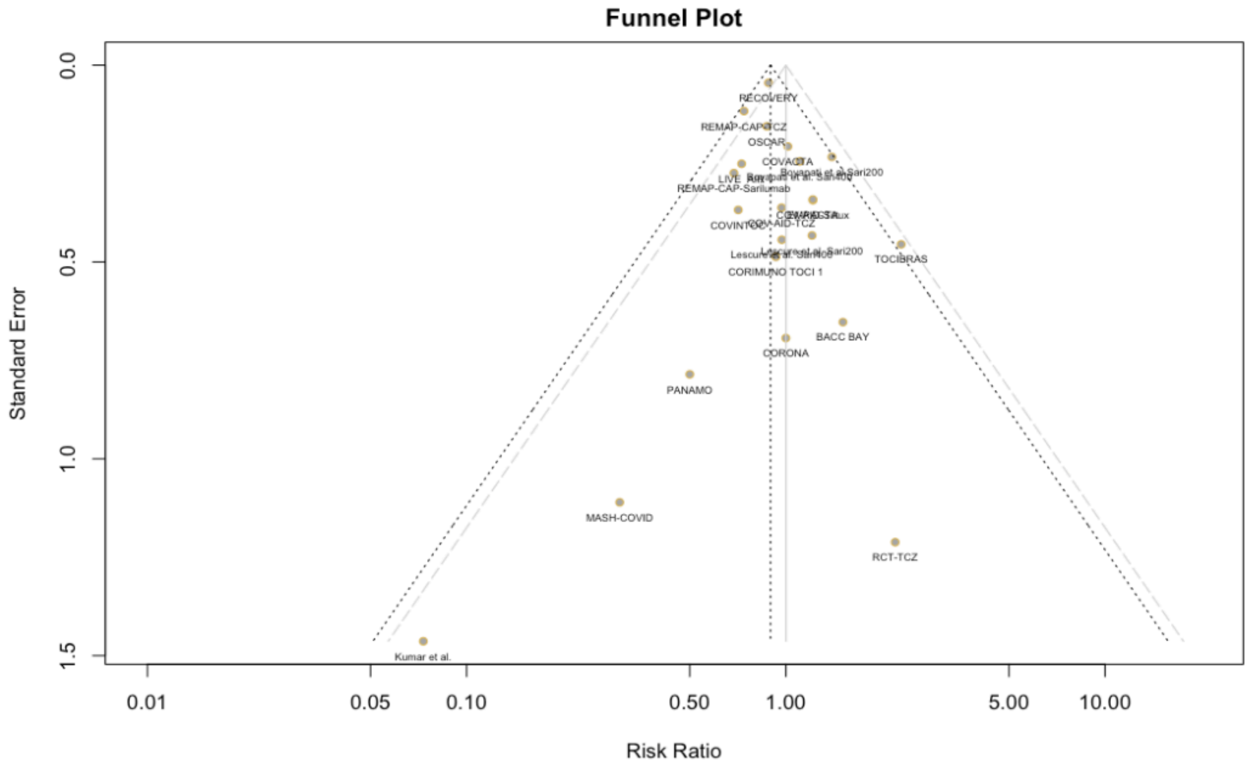

Appendix, Figure 3: Funnel plot for demonstrating publication bias.

The distribution of included studies is relatively symmetrical, suggesting no presence of publication bias.

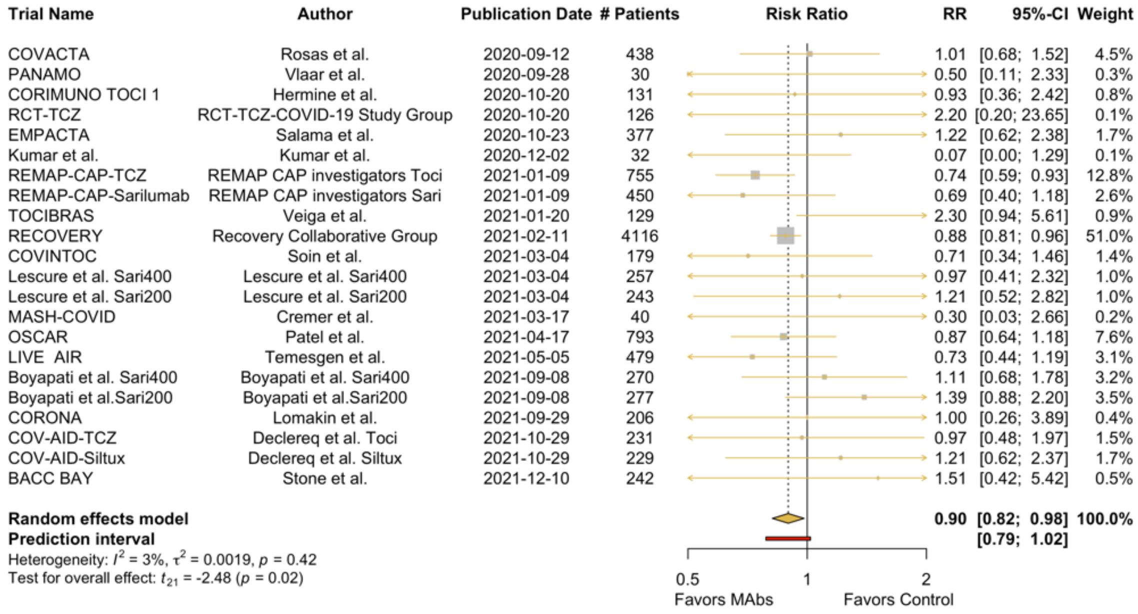

Appendix, Figure 4: Traditional (non-cumulative) meta-analysis Forest plot.

95% confidence interval, proportion of patients with severe disease at baseline and overall pooled effect size.

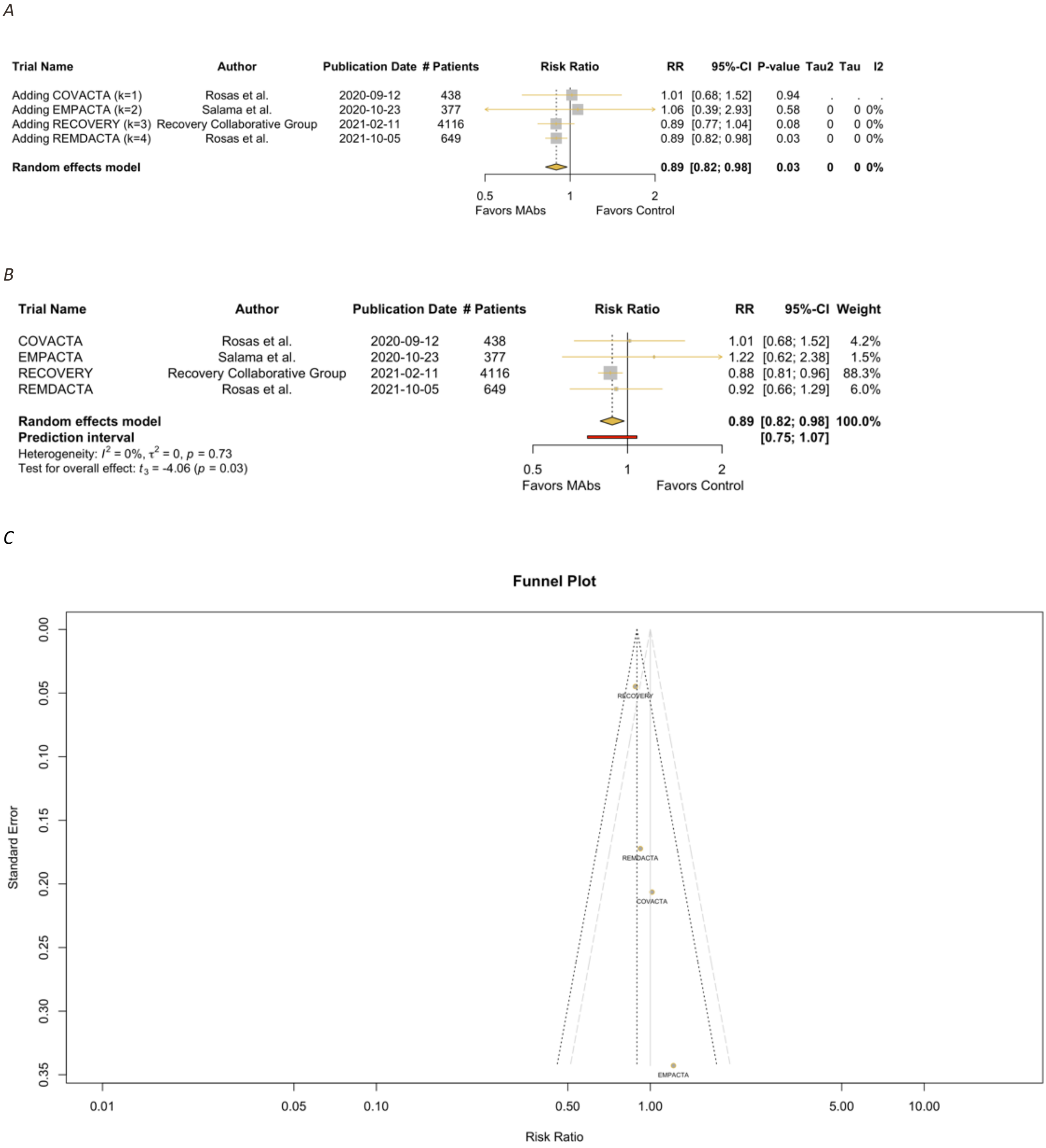

Appendix, Figure 5: Subset FDA trials

A: Sub-group cumulative meta-analysis that only includes the 4 trials named by the FDA as trials that influenced their decision for EUA and Approval. Note that the 4th trial added, REMDACTA, is not included in our cumulative meta-analysis, as the publication was not retrieved on the date of our literature search.

B: Traditional (non-cumulative) meta-analysis showing the contribution of each individual study to the overall estimate

C: Funnel plot that shows an asymmetric distribution of included paper in the analysis, suggesting publication bias. Note that the included studies are not claimed to have been systematically included by the FDA.

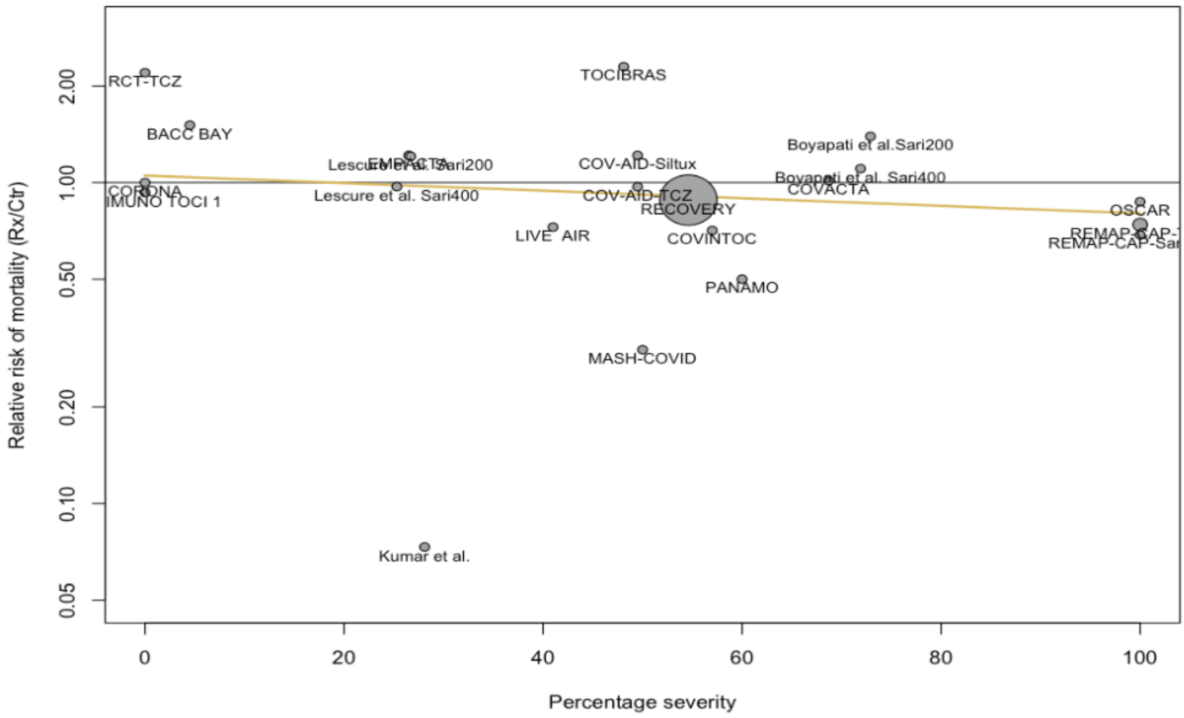

Appendix, Figure 6: Meta-regression of Severity.

Investigating the impact of the % of severe patients in the study versus the RR of mortality in the intervention/control arm. Note that this meta-analysis is performed with aggregate data, which is considered less reliable than with individual level data.

Decision-analytic model

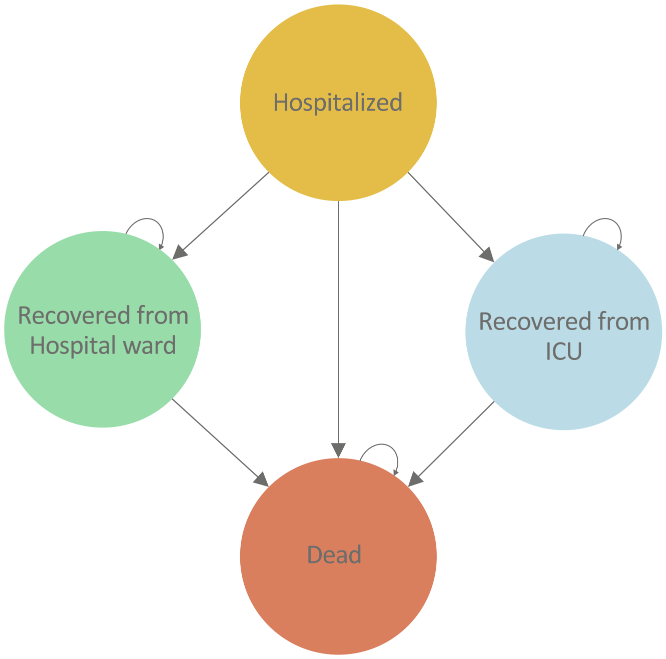

Appendix, Figure 7: State-transition diagram.

The model states are: Hospitalized, Recovered from the Hospital Ward as the highest level of care, Recovered from the ICU as the highest level of care or Dead. The arrows indicate the potential transitions

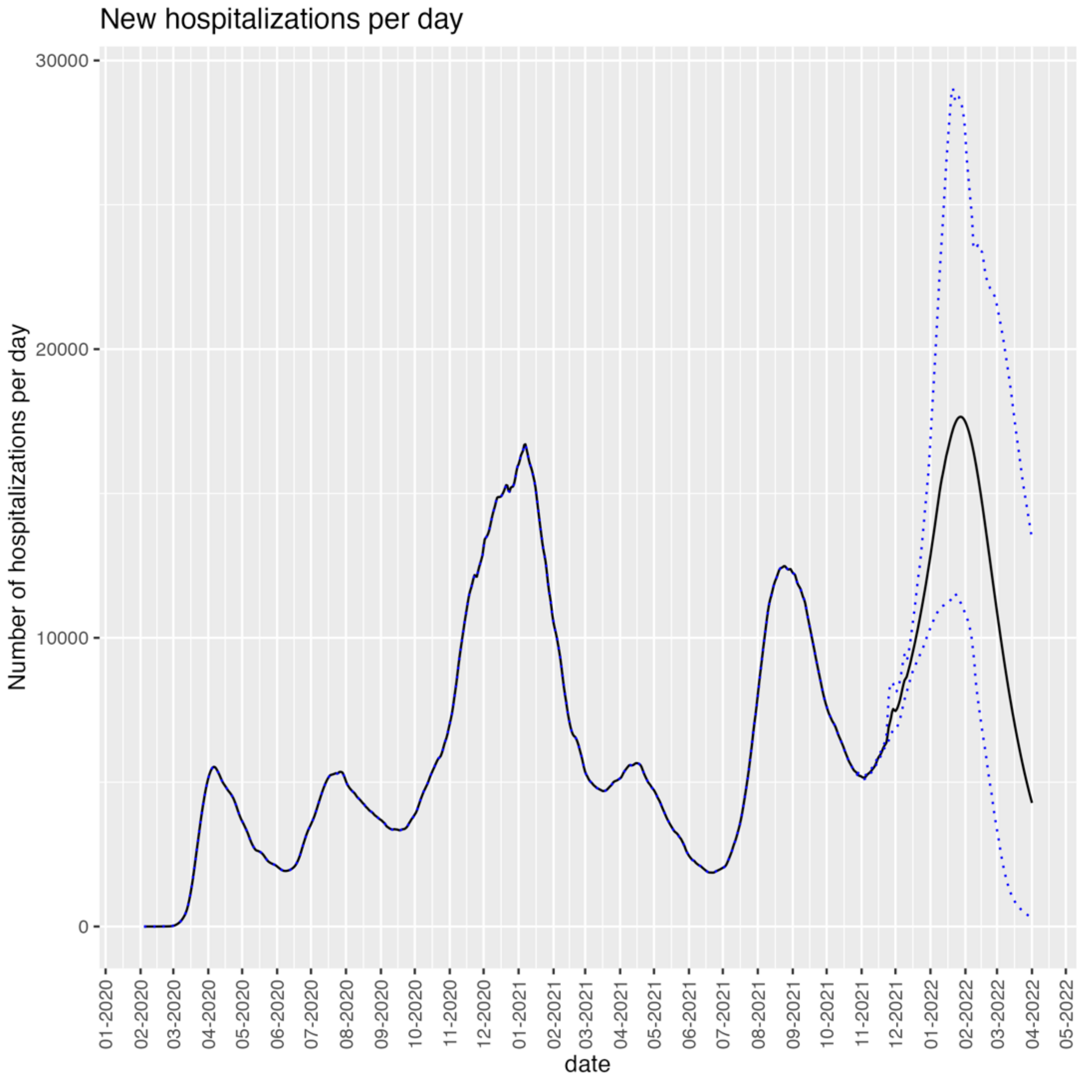

Appendix, Figure 8: IHME predictions – Retrospective.

Forecasted number of patients hospitalized per day used in retrospective VOI analysis. This graph is predicted on the last dataset published (19 December 2023) prior to the end-date of our timeline (01 January 2022).

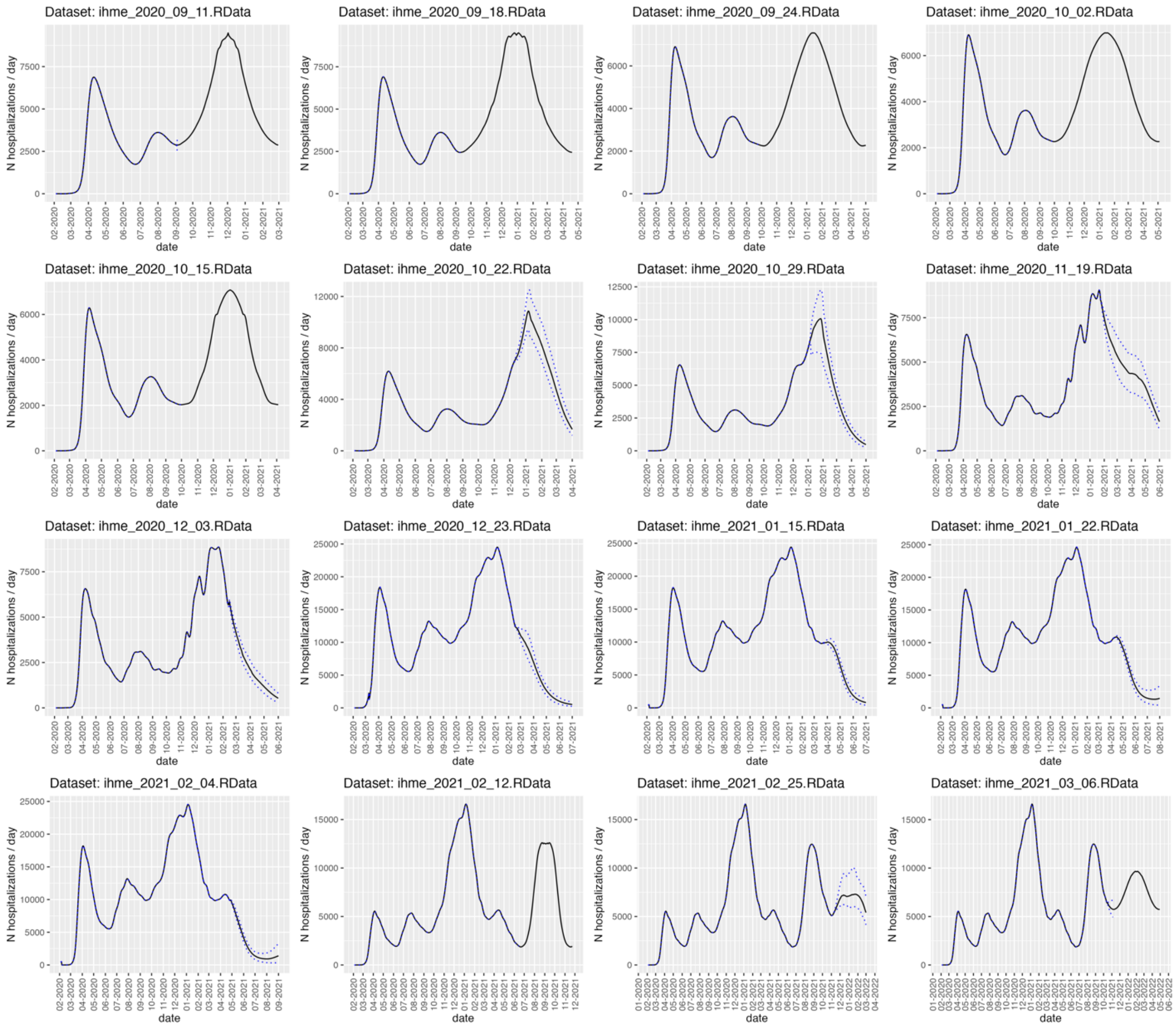

Appendix, Figure 9: IHME predictions – Prospective.

Forecasted number of projected hospitalizations as projected at various dates across time used in the prospective VOI analysis.

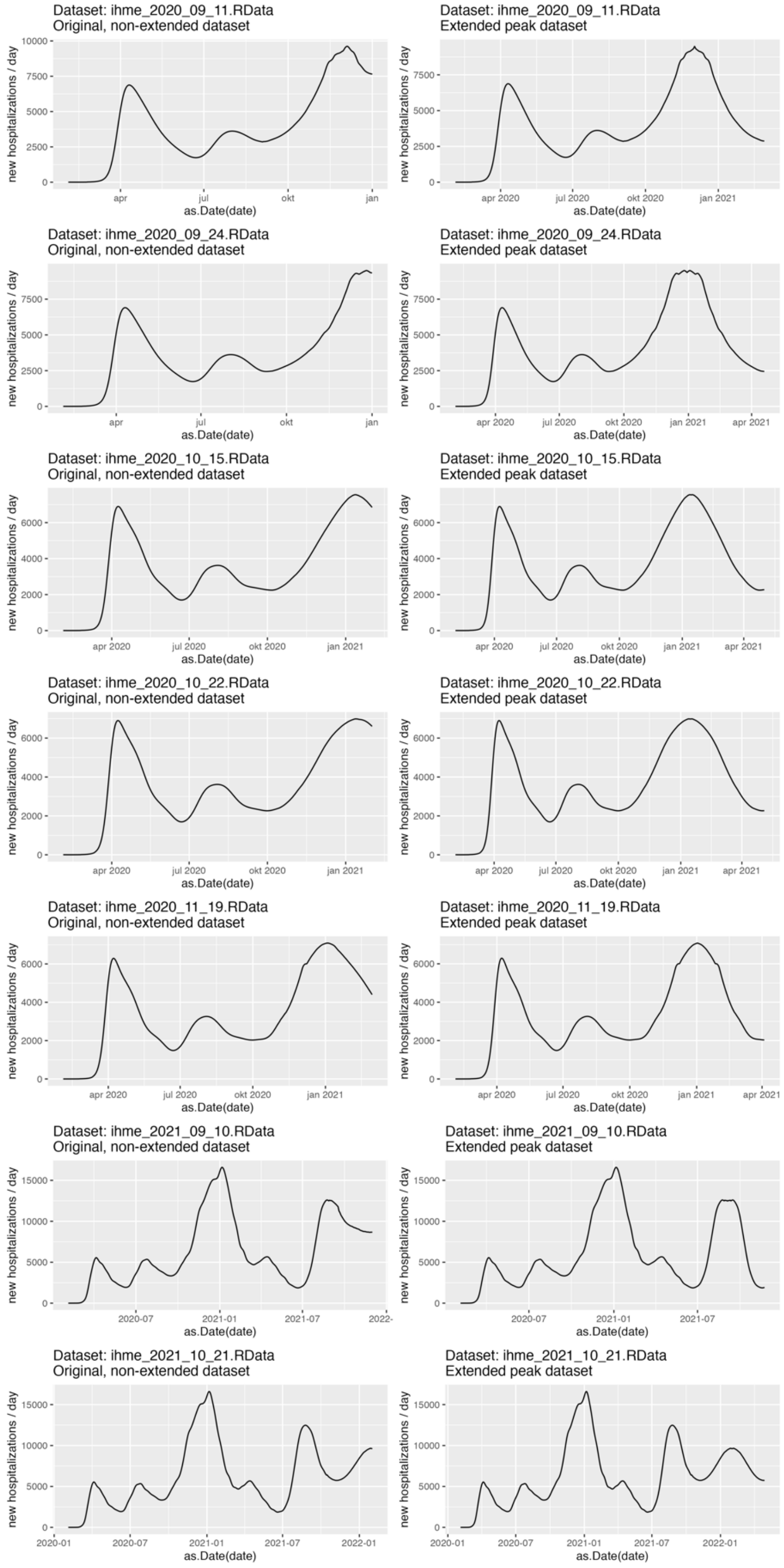

Appendix, Figure 10: Extended and non-extended peak IHME predictions.

On the left side we show the original, unadjusted IHME datasets that we selected ended mid-peak. On the right side we show the in the analysis extended peaks used for our main analysis, which are the versions also used in the previous figure. In a sensitivity analysis we show the results for the non-extended peak data.

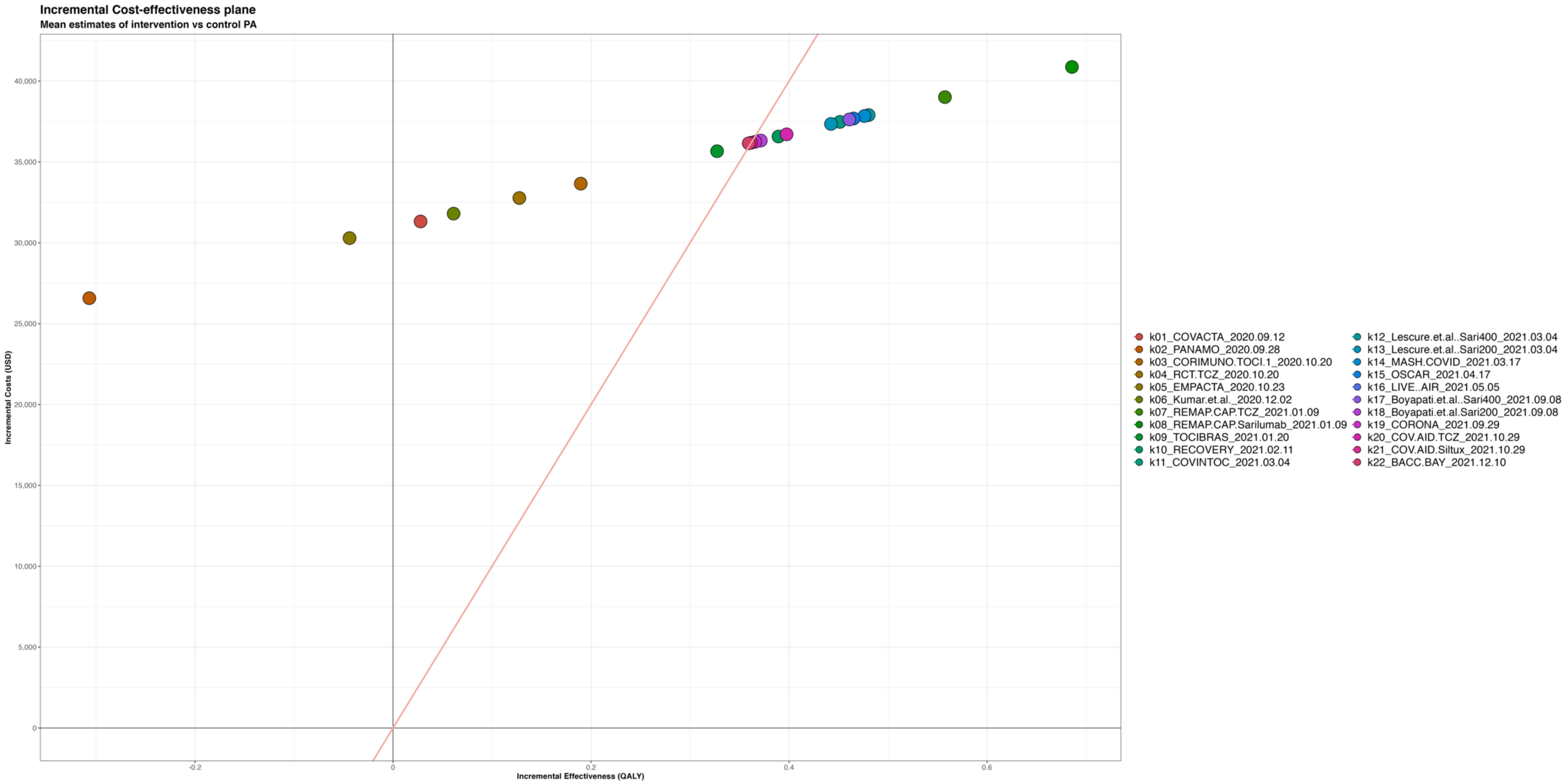

Appendix, Figure 11: Incremental Cost-effectiveness planes (Rx vs Cx) for all timepoints using cumulative meta-analysis results.

The figure shows the incremental cost-effectiveness planes across time, with each circle representing the cumulative evidence up until and including that trial. The figure shows the CE plane for QALY versus cost in USD. The line shows the WTP line of $100.000/QALY, all studies falling below this line are considered cost-effective. The figure is mainly indicative of the changes in cost-effectiveness over time.

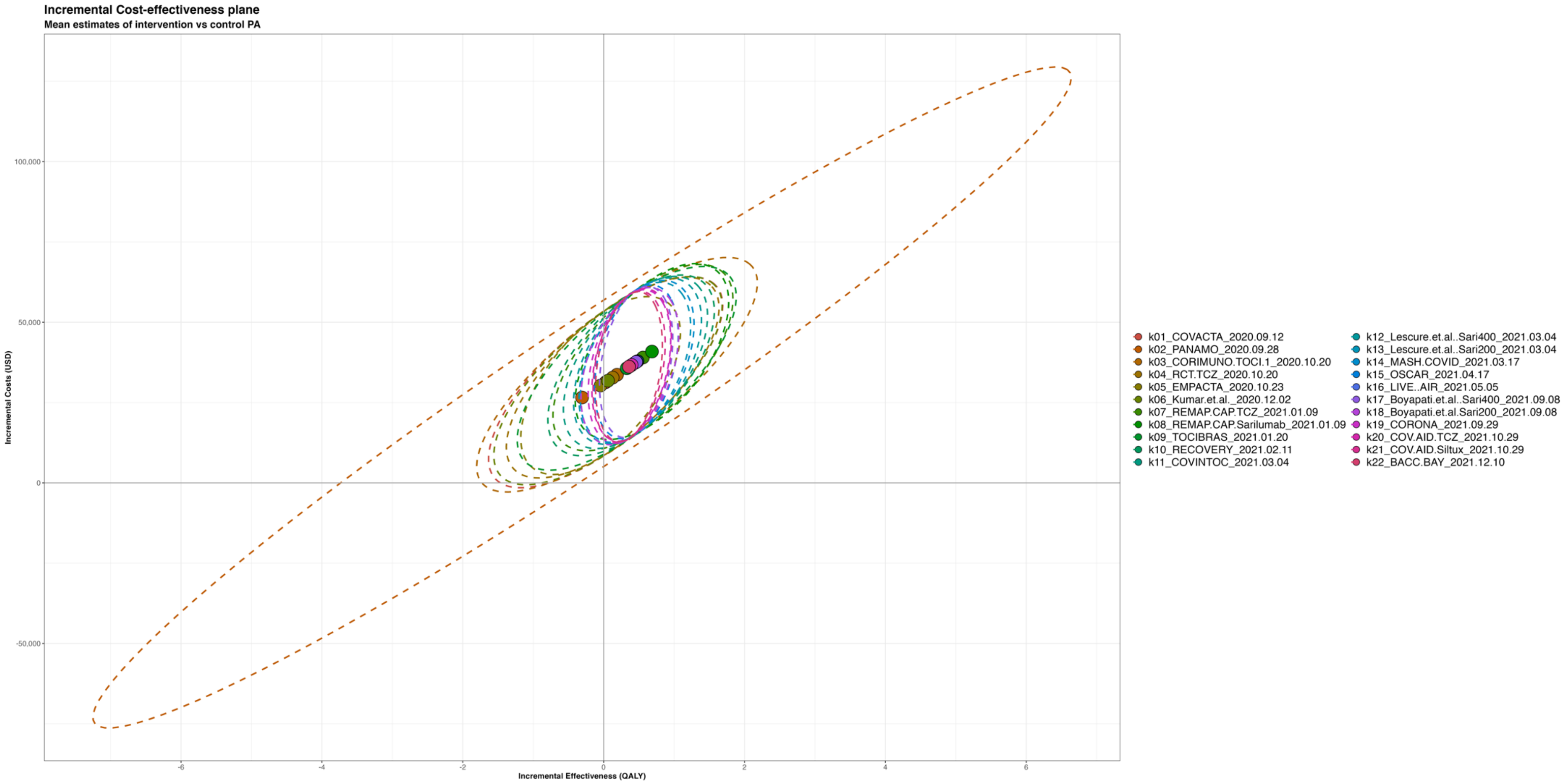

Appendix, Figure 12: Incremental Cost-effectiveness planes (Rx vs Cx) for all timepoints using cumulative meta-analysis results with uncertainty ellipse.

The figure shows the incremental cost-effectiveness planes across time, with each circle representing the cumulative evidence up until and including that trial. The figure shows the CE plane for QALY versus cost in USD. The line shows the WTP line of $100.000/QALY, all studies falling below this line are considered cost-effective. The figure is mainly indicative of the changes in cost-effectiveness over time. The ellipses around the studies indicate 95% Credibility Intervals.

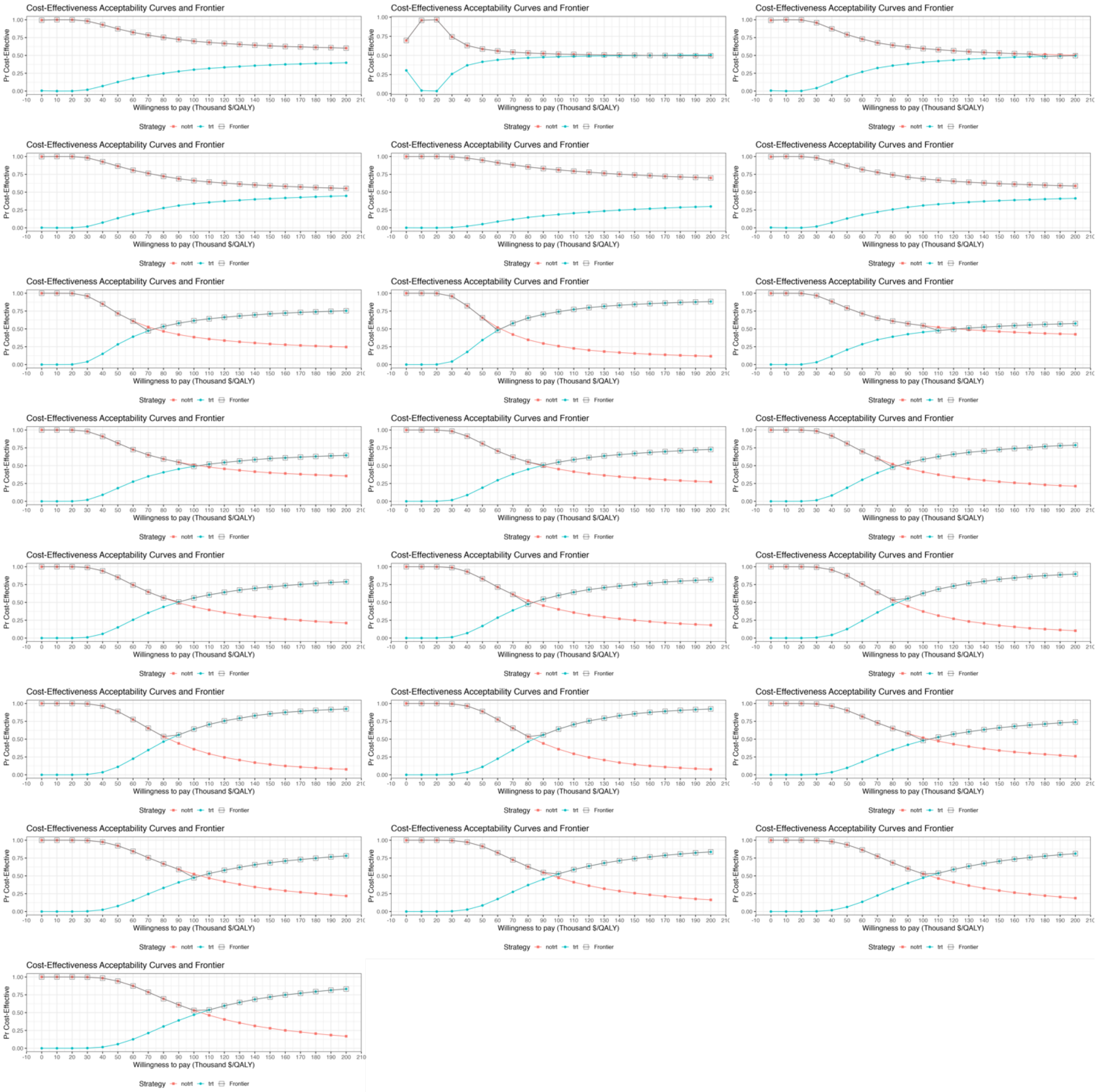

Appendix, Figure 13: Cost-Effectiveness Curves and Frontiers.

The figures show the probability of treatment with Monoclonal Antibodies (trt, blue) and no treatment (notrt, red) on the y-axis across a range of WTP thresholds (x-axis). The treatment on the Frontier is indicated with a box surrounding the estimates on the curve.

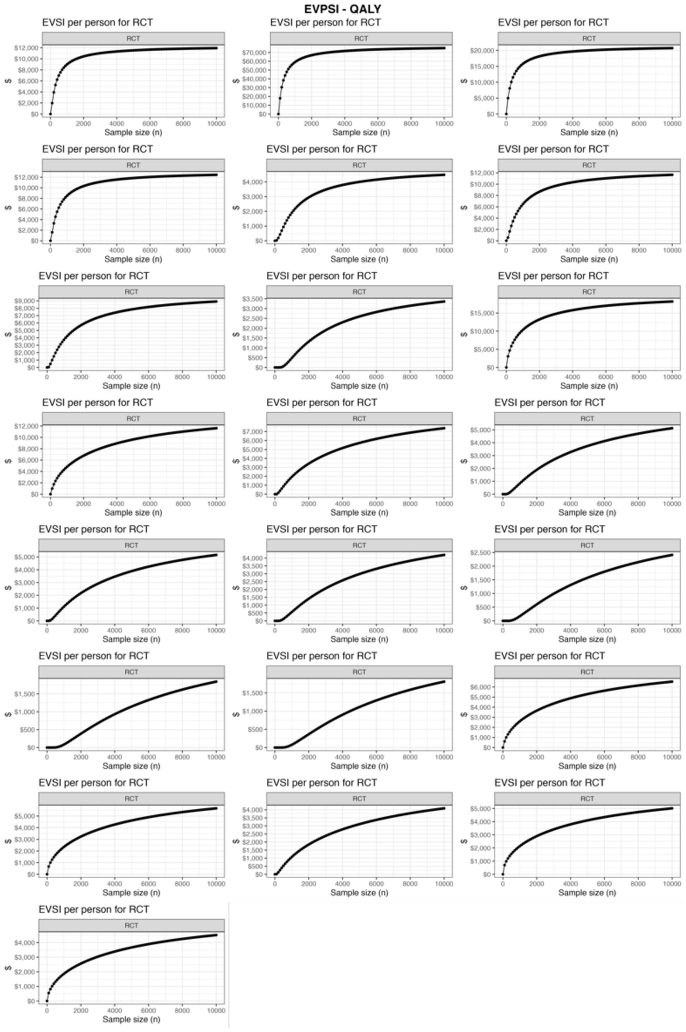

A

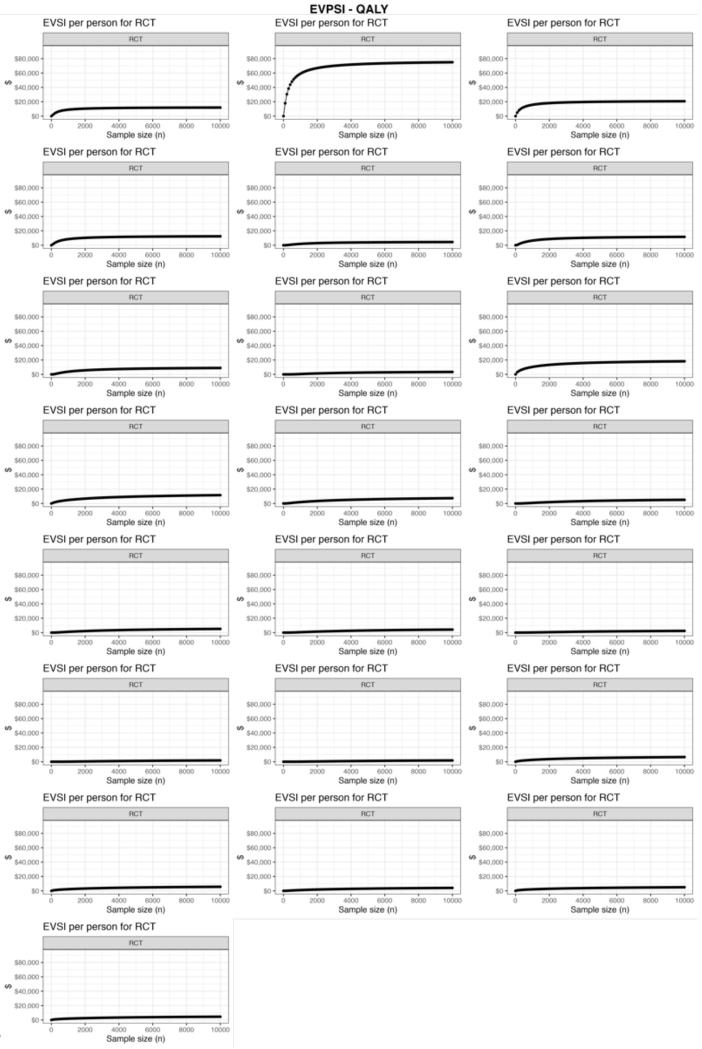

B

Appendix, Figure 14. EVPSI plots (QALY) over time.

EVPSI in USD is shown on the Y axis, sample size on the X axis. Figure A shows the EVPSI plots zoomed in with varying xy-limits to read off the individual costs, Figure B shows the same figure but with set x and y limits to show the relative progression of over time. In each plot we see that with an increasing sample size we see that the additional value obtained from additional person decreases.

	k01_COVACTA	k02_PANAMO	k03_CORIMUNO TOCI 1	k04_RCT-TCZ	k05_EMPACTA	k06_Kumar et al.	k07_REMAP-CAP-TCZ	k08_REMAP-CAP-Sarilumab	k09_TOCIBRAS	k10_RECOVERY	k11_COVINTOC	k12_Lescure et al. Sari400	k13_Lescure et al. Sari200	k14_MASH-COVID	k15_OSCAR	k16_LIVE AIR	k17_Boyapati et al. Sari400	k18_Boyapati et al.Sari200	k19_CORONA	k20_COV-AID-TCZ	k21_COV-AID-Siltux	k22_BACC BAY
Cost-effective	No	No*	No	No	No*	No	Yes	Yes	No	Yes	Yes	Yes	Yes	Yes	Yes	Yes	Yes	Yes	Yes	Yes	No	No
Incr cost Rx	31319.14	26576.61	33658.91	32769.56	30291.4	31804.06	39008.52	40872.95	35664.55	36572.18	37475.53	37898.92	37350.36	37839.44	37687.12	37625.34	37625.34	36325.26	36245.59	36706.64	36185.75	36153.7
Incr effect Rx	0.03	-0.31	0.19	0.13	-0.04	0.06	0.56	0.69	0.33	0.39	0.45	0.48	0.44	0.48	0.47	0.46	0.46	0.37	0.37	0.4	0.36	0.36
ICER	1126586.47	n/a	177503.43	256840.7	n/a	519872.93	69977.62	59607.1	108971.91	93944.79	83032.93	78900	84440.57	79482.12	81009.71	81646.69	81646.69	97785.46	99084.3	92329.89	100083.67	100627.94
Incr NMB	-28539.14	-57252.29	-14696.51	-20010.85	-34679.58	-25686.4	16735.76	27697.65	-2936.35	2357.26	7657.8	10135.2	6882.36	9768.05	8834.61	8457.78	8457.78	822.65	334.97	3049.33	-30.25	-225.61
Incr NHB	-0.29	-0.57	-0.15	-0.2	-0.35	-0.26	0.17	0.28	-0.03	0.02	0.08	0.1	0.07	0.1	0.09	0.08	0.08	0.01	0	0.03	0	0
EVPPI (Million)	8542	69270	15235	9304	3354	3479	1430	620	1266	2910	330	247	241	420	161	203	215	568	212	6609	7438	1771
Future patients (Thousand)	690	915	708	708	663	273	139	139	62	190	31	31	31	63	38	58	62	62	27	1054	1054	275
Current patients (Thousand)	290	253	245	245	274	400	530	530	400	324	156	156	156	389	388	336	252	252	462	387	387	441
Optimal strategy	OIR	OIR	OIR	OIR	OIR	OIR	AWR	AWR	OIR	AWR	AWR	AWR	AWR	AWR	Approve	Approve	Approve	AWR	AWR	AWR	OIR	OIR
Optimal sample size (N)*	10000	10000	10000	10000	10000	10000	9900	6900	10000	10000	6200	4800	5000	8000	n/a	n/a	n/a	10000	4700	10000	10000	10000
Net value for:	n/a	n/a	n/a	n/a	n/a	n/a	n/a	n/a	n/a	n/a	n/a	n/a	n/a	n/a	n/a	n/a	n/a	n/a	n/a	n/a	n/a	n/a
OIR (Million)	7999	68254	14512	8629	2703	2960	3556	4365	1013	2573	412	432	315	829	371	547	580	362	79	7440	5186	1145
AWR (Million)	-19695	1921	651	-10244	-29446	-14080	12266	18798	-314	3313	1543	1937	1340	4534	3752	3332	2645	560	233	8591	5143	985
Approve (Million)	-27979	-66905	-14008	-19073	-32496	-17298	11207	18548	-1357	1213	1434	1898	1289	4413	3762	3339	2650	258	164	4395	-44	-162
Reject	0	0	0	0	0	0	0	0	0	0	0	0	0	0	0	0	0	0	0	0	0	0
Equation parameters:	n/a	n/a	n/a	n/a	n/a	n/a	n/a	n/a	n/a	n/a	n/a	n/a	n/a	n/a	n/a	n/a	n/a	n/a	n/a	n/a	n/a	n/a
Costs RCT (Million)	97	97	97	97	97	97	96	70	97	97	64	52	54	80	n/a	n/a	n/a	97	51	97	97	97
*EVSI N**	11938	74986	20723	12457	4485	11654	8892	2984	18173	11612	6271	3654	3890	3825	n/a	n/a	n/a	6529	4518	4088	5014	4519
Below values for sample size of N = 2500	n/a	n/a	n/a	n/a	n/a	n/a	n/a	n/a	n/a	n/a	n/a	n/a	n/a	n/a	n/a	n/a	n/a	n/a	n/a	n/a	n/a	n/a
Costs RCT N=2500 (Million)	32	32	32	32	32	32	32	32	32	32	32	32	32	32	n/a	32	32	32	32	32	32	32
EVSI N=2500	10778	68721	18720	10790	3251	9275	6281	1635	14119	7468	3963	2309	2566	1762	n/a	537	514	4037	3553	2135	3176	2818
AWR N=2500 (Million)	-20537	-3962	-759	-11436	-30329	-14762	12029	18709	-511	2599	1517	1926	1329	4479	n/a	3328	2640	474	226	6610	3271	582
OIR N=2500 (Million)	7371	62799	13212	7588	2080	2471	3193	4086	839	1840	342	370	273	705	n/a	504	533	270	72	5436	3315	743

Appendix, Figure 15: Prospective analysis, full results table.

The columns represent the various timepoints (new studies added), the rows indicate which results are represented. Reading the table from left to right shows the changes in values over time. The "reject" strategy results in continuing care as usual and is considered the reference strategy. Adopting this strategy results in an expected value of $0.

	k01_COVACTA	k02_PANAMO	k03_CORIMUNO TOCI 1	k04_RCT-TCZ	k05_EMPACTA	k06_Kumar et al.	k07_REMAP-CAP-TCZ	k08_REMAP-CAP-Sarilumab	k09_TOCIBRAS	k10_RECOVERY	k11_COVINTOC	k12_Lescure et al. Sari400	k13_Lescure et al. Sari200	k14_MASH-COVID	k15_OSCAR	k16_LIVE AIR	k17_Boyapati et al. Sari400	k18_Boyapati et al.Sari200	k19_CORONA	k20_COV-AID-TCZ	k21_COV-AID-Siltux	k22_BACC BAY
Cost-effective	No	No*	No	No	No*	No	Yes	Yes	No	Yes	Yes	Yes	Yes	Yes	Yes	Yes	Yes	Yes	Yes	Yes	No	No
Incr cost Rx	31319.14	26576.61	33658.91	32769.56	30291.4	31804.06	39008.52	40872.95	35664.55	36572.18	37475.53	37898.92	37350.36	37839.44	37687.12	37625.34	37625.34	36325.26	36245.59	36706.64	36185.75	36153.7
Incr effect Rx	0.03	-0.31	0.19	0.13	-0.04	0.06	0.56	0.69	0.33	0.39	0.45	0.48	0.44	0.48	0.47	0.46	0.46	0.37	0.37	0.4	0.36	0.36
ICER	1126586.47	n/a	177503.43	256840.7	n/a	519872.93	69977.62	59607.1	108971.91	93944.79	83032.93	78900	84440.57	79482.12	81009.71	81646.69	81646.69	97785.46	99084.3	92329.89	100083.67	100627.94
Incr NMB	-28539.14	-57252.29	-14696.51	-20010.85	-34679.58	-25686.4	16735.76	27697.65	-2936.35	2357.26	7657.8	10135.2	6882.36	9768.05	8834.61	8457.78	8457.78	822.65	334.97	3049.33	-30.25	-225.61
Incr NHB	-0.29	-0.57	-0.15	-0.2	-0.35	-0.26	0.17	0.28	-0.03	0.02	0.08	0.1	0.07	0.1	0.09	0.08	0.08	0.01	0	0.03	0	0
EVPPI (Million)	54781	321051	84567	51647	19668	41848	31267	13551	61134	43914	29021	21735	21177	18070	11207	9004	5507	14558	11549	7391	8318	3168
Future patients (Thousand)	4426	4242	3933	3933	3887	3288	3043	3043	2991	2872	2757	2757	2757	2705	2625	2590	1584	1584	1456	1178	1178	492
Current patients (Thousand)	331	462	673	673	702	913	579	579	462	337	316	316	316	303	221	165	460	460	382	470	470	893
Optimal strategy	OIR	OIR	OIR	OIR	OIR	OIR	AWR	AWR	OIR	AWR	AWR	AWR	AWR	AWR	AWR	AWR	AWR	AWR	AWR	AWR	OIR	OIR
Optimal sample size (N)*	10000	10000	10000	10000	10000	10000	10000	10000	10000	10000	10000	10000	10000	10000	10000	10000	10000	10000	10000	10000	10000	10000
Net value for:	n/a	n/a	n/a	n/a	n/a	n/a	n/a	n/a	n/a	n/a	n/a	n/a	n/a	n/a	n/a	n/a	n/a	n/a	n/a	n/a	n/a	n/a
OIR (Million)	52596	317733	81332	48796	17164	38100	78006	94538	54234	40030	41387	42016	33129	37729	29465	26614	16209	11552	8628	8329	5811	2125
AWR (Million)	-82869	48949	13787	-43173	-141621	-69562	87537	110310	44127	40801	43728	45115	35232	40591	31327	27923	20018	11923	8753	9731	5762	1815
Approve (Million)	-135751	-269357	-67692	-92169	-159132	-107919	60622	100330	-10136	7565	23528	31139	21145	29383	25139	23297	17291	1682	615	5026	-50	-313
Reject	0	0	0	0	0	0	0	0	0	0	0	0	0	0	0	0	0	0	0	0	0	0
Equation parameters:	n/a	n/a	n/a	n/a	n/a	n/a	n/a	n/a	n/a	n/a	n/a	n/a	n/a	n/a	n/a	n/a	n/a	n/a	n/a	n/a	n/a	n/a
Costs RCT (Million)	97	97	97	97	97	97	97	97	97	97	97	97	97	97	97	97	97	97	97	97	97	97
*EVSI N**	11938	74986	20723	12457	4485	11654	8904	3357	18173	11612	7377	5123	5158	4197	2411	1840	1810	6529	5657	4088	5014	4519
Below values for sample size of N = 2500	n/a	n/a	n/a	n/a	n/a	n/a	n/a	n/a	n/a	n/a	n/a	n/a	n/a	n/a	n/a	n/a	n/a	n/a	n/a	n/a	n/a	n/a
Costs RCT N=2500 (Million)	32	32	32	32	32	32	32	32	32	32	32	32	32	32	32	32	32	32	32	32	32	32
EVSI N=2500	10778	68721	18720	10790	3251	9275	6281	1635	14119	7468	3963	2309	2566	1762	804	537	514	4037	3553	2135	3176	2818
AWR N=2500 (Million)	-88042	22223	5916	-49742	-146483	-77419	79682	105238	32059	28974	34411	37460	28177	34105	27208	24643	18062	8043	5755	7507	3661	1043
OIR N=2500 (Million)	47637	291437	73571	42377	12562	30436	70026	89257	42188	28185	32013	34286	26022	31170	25280	23272	14190	7666	5628	6082	3711	1355

Appendix, Figure 16: Retrospective analysis, full results table.

The columns represent the various timepoints (new studies added), the rows indicate which results are represented. Reading the table from left to right shows the changes in values over time. The "reject" strategy results in continuing care as usual and is considered the reference strategy. Adopting this strategy results in an expected value of $0.

Sensitivity analyses

A

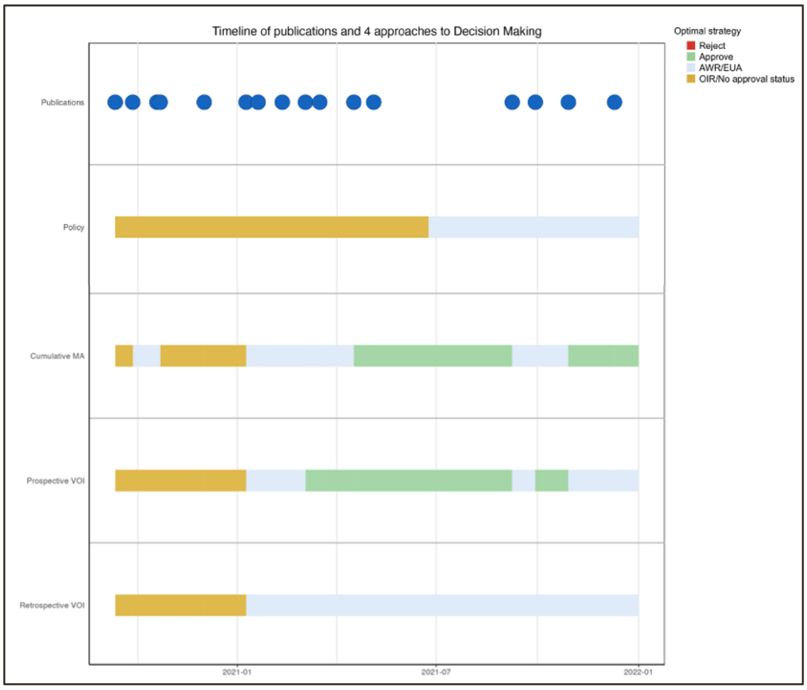

B

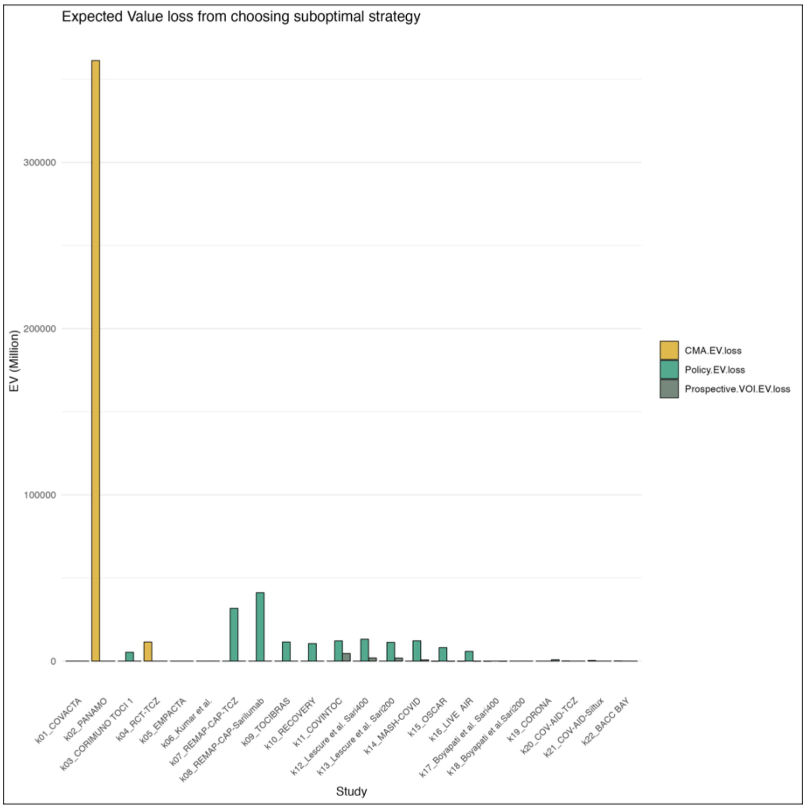

Appendix, Table 5: Sensitivity Analysis – WTP $150.000.

A: Timeline results. With a higher WTP than the main analysis (WTP $100.000), K9 that was previously not considered cost-effective now is. Prospective VOI favored approval for a longer period (now K11-K17+K19, previously K15-K17), but retrospective VOI remained unchanged in which strategies had the highest total Expected Net Benefit.

B: Loss figure comparing approach strategy to retrospective optimal strategy. Loss increased for the Prospective VOI when Approval was the suggested

A

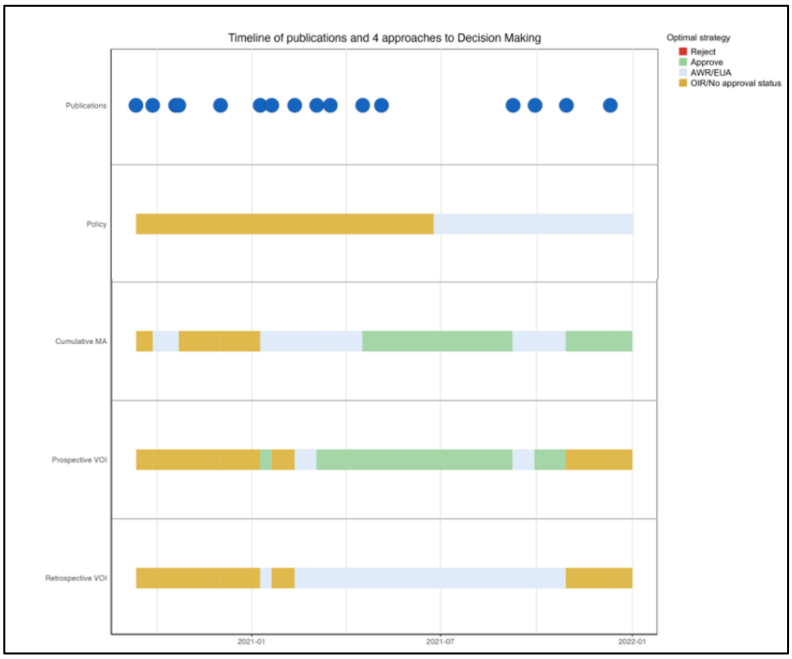

B

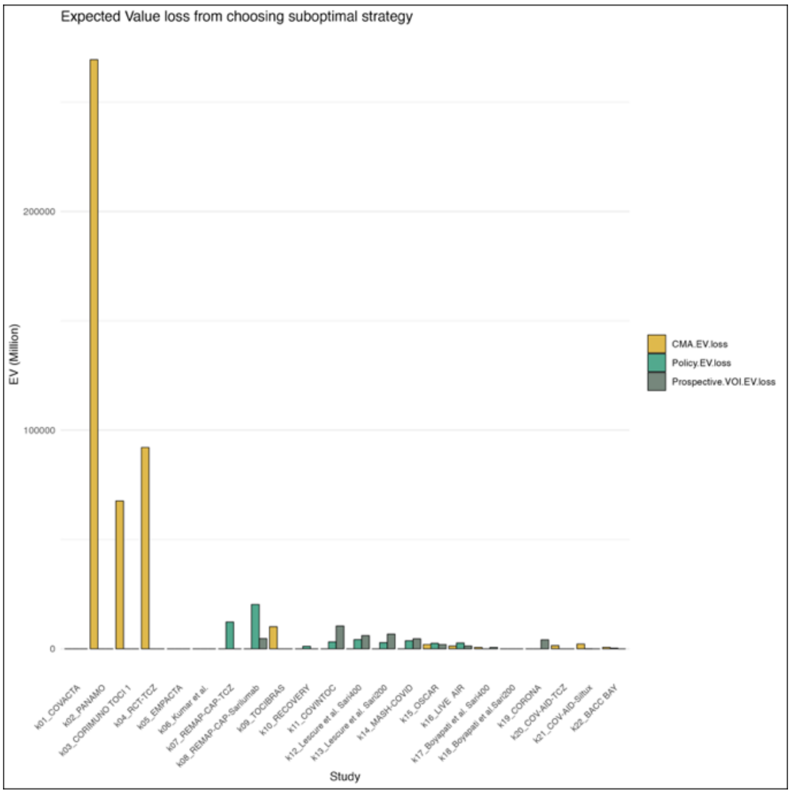

Appendix, Table 6: Sensitivity Analysis – 3-month trial duration

A: Timeline results. Prospective VOI suggests approval as the optimal strategy more frequently than with a 3-month trial duration (opposed to the 2-month trial duration in the main analysis). This can be attributed to the low number of patients in the category "future patients" as the IHME predictions only last shortly into the future. For example, the number of future patients calculated in K11-K13, K15 and K19 are only between 1-3 thousand. If very few patients can benefit from future research, even when EVPPI is high, the costs of a new trial do not outweigh the benefits.
B: Loss figure comparing approach strategy to retrospective optimal strategy. The loss from the prospective VOI approach is greater than in the main analysis as Approval is more frequently suggested

A

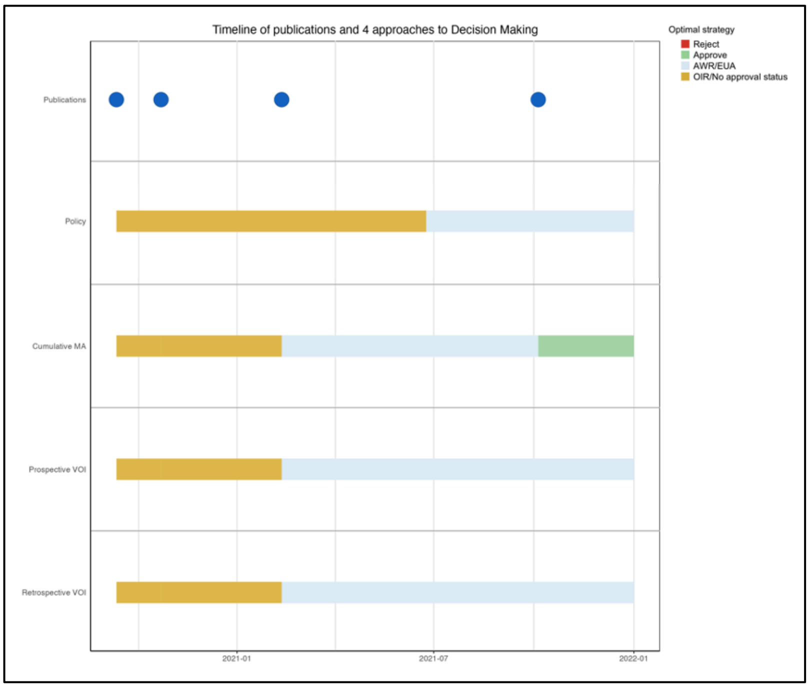

B

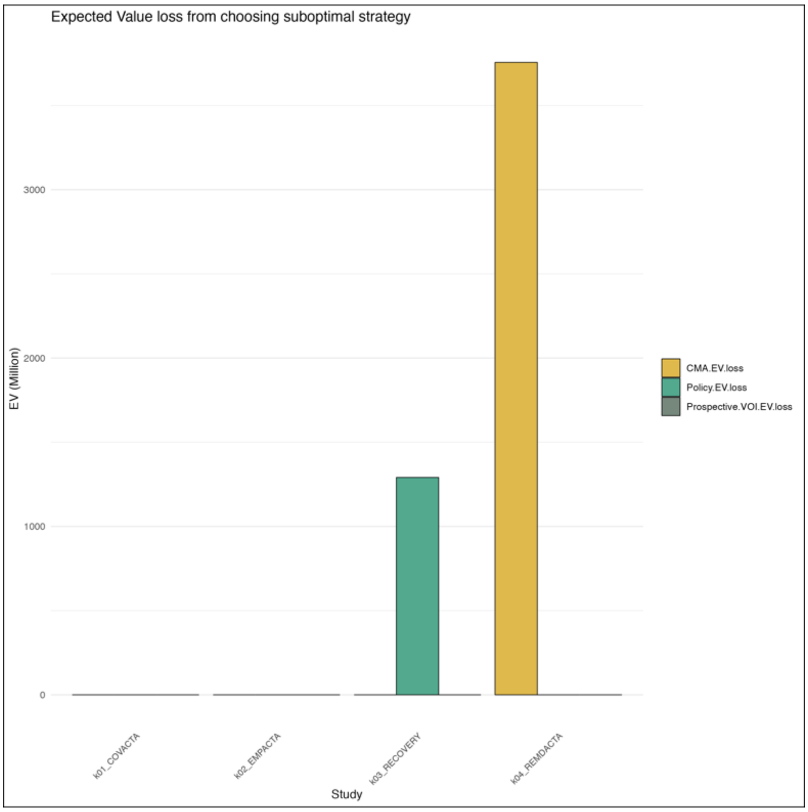

Appendix, Table 7: Sensitivity Analysis – FDA included trials only

A: Timeline results. The timeline figure shows that FDA's decision for EUA and Approval were inconsistent with both CMA and VOI or could be interpreted as delayed from the CMA results. VOI both prospectively and retrospectively suggest OIR followed by AWR, but never Approval or Rejection

B: Loss figure comparing approach strategy to retrospective optimal strategy. This expresses the loss encountered from the inconsistency between the VOI approaches after K3.

A

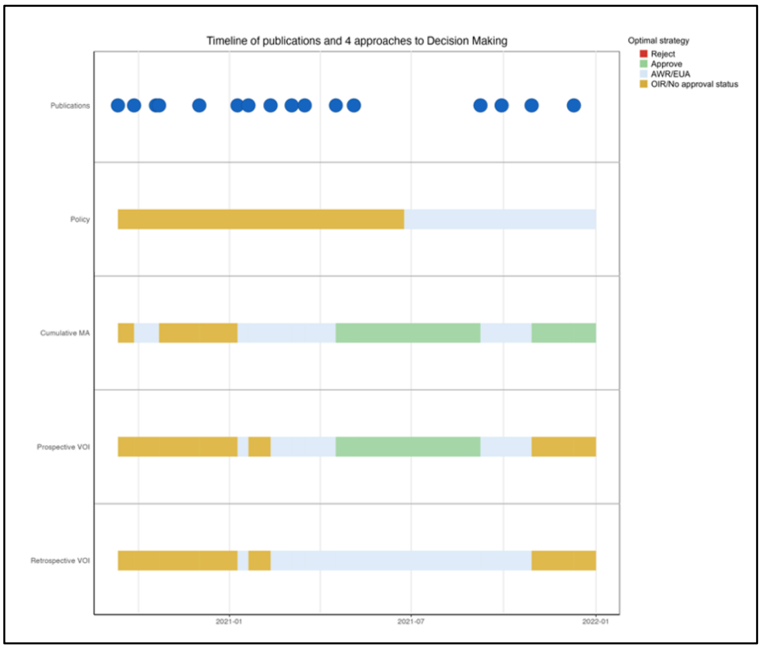

B

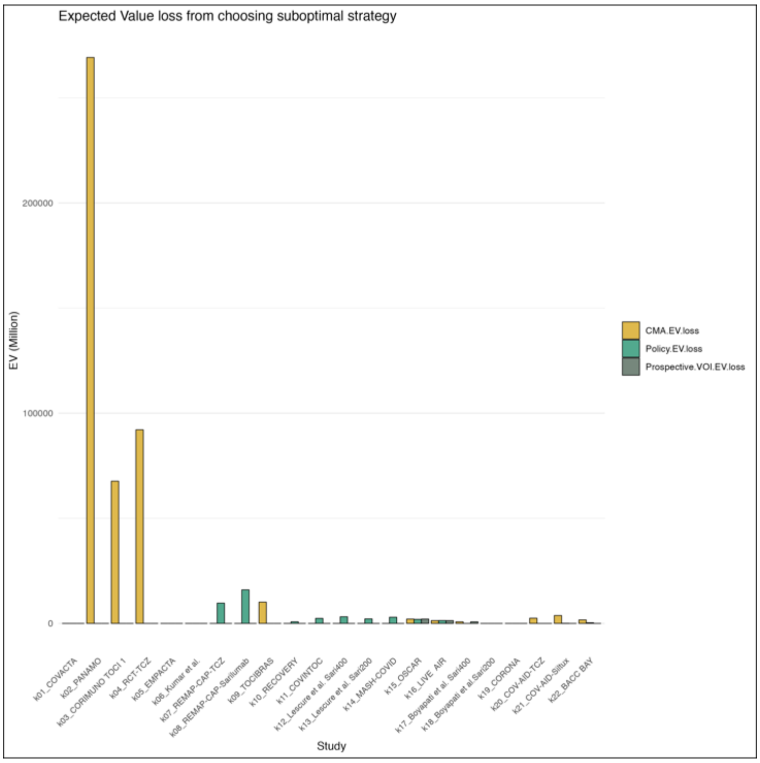

Appendix, Table 8: Sensitivity Analysis – No extended peaks (unadjusted IHME data)

A: Timeline results. The extended peaks in our main analysis did not influence which strategy held the highest EV for either VOI approach.

B: Loss figure comparing approach strategy to retrospective optimal strategy. As there were fewer patients who benefit from research, EV was lower without peak extension for those timepoints when they were applicable

A

B

C

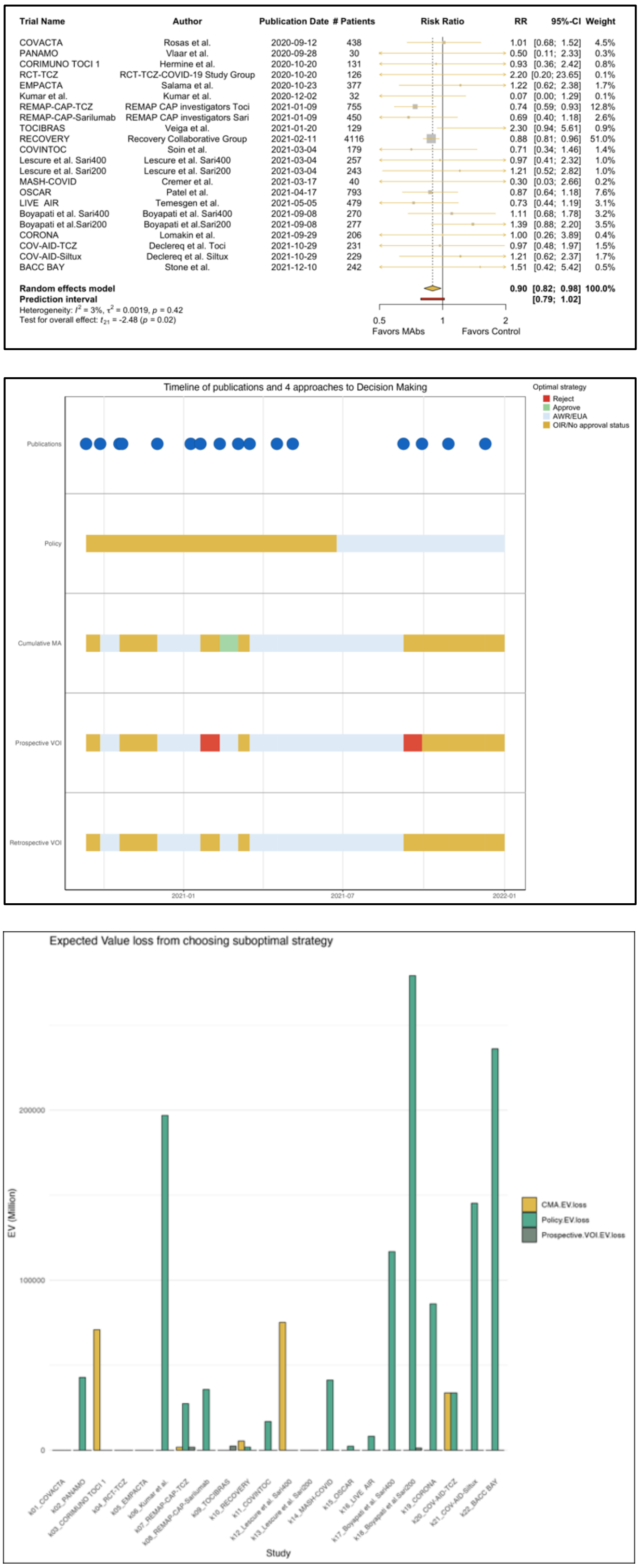

Appendix, Table 9: Sensitivity Analysis – Traditional meta-analysis (non-cumulative)

A: Forest plot: The non-cumulative meta-analysis forest plot shows the individual contribution of each study to the meta-analysis.

B: Timeline results. The timeline shows now the results of the optimal suggested strategy based on each approach if only single studies were used in the model.

C: Loss figure comparing approach strategy to retrospective optimal strategy

A

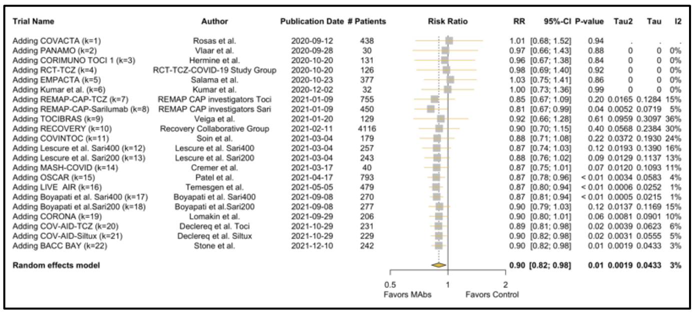

B

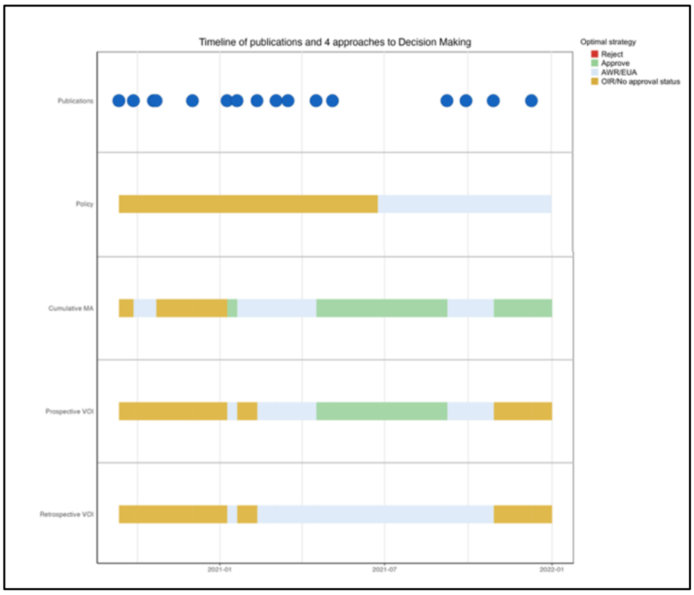

C

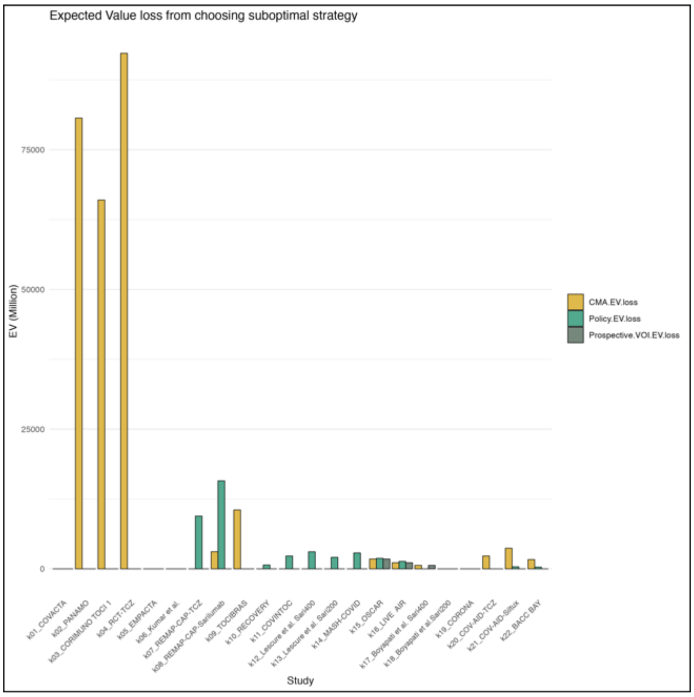

Appendix, Table 10: Sensitivity Analysis – Basic CMA (no Knapp Hartung adjustment)

A: Forest plot. Not applying the Knapp Hartung adjustment in the meta-analysis results in smaller confidence intervals surrounding the Relative Risk estimates.

B: Timeline results. Approval is already suggested at an earlier stage in the CMA.

C: Loss figure comparing approach strategy to retrospective optimal strategy. As there is less uncertainty, EVPPI is smaller, and loss from suboptimal strategies is as well

A

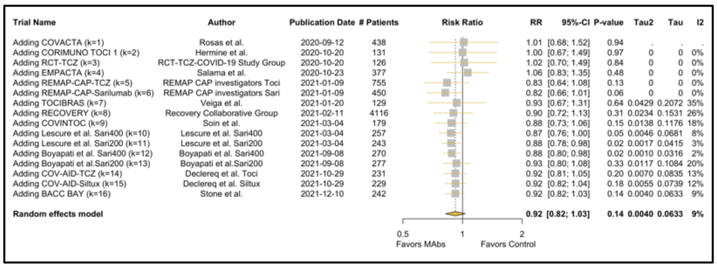

B

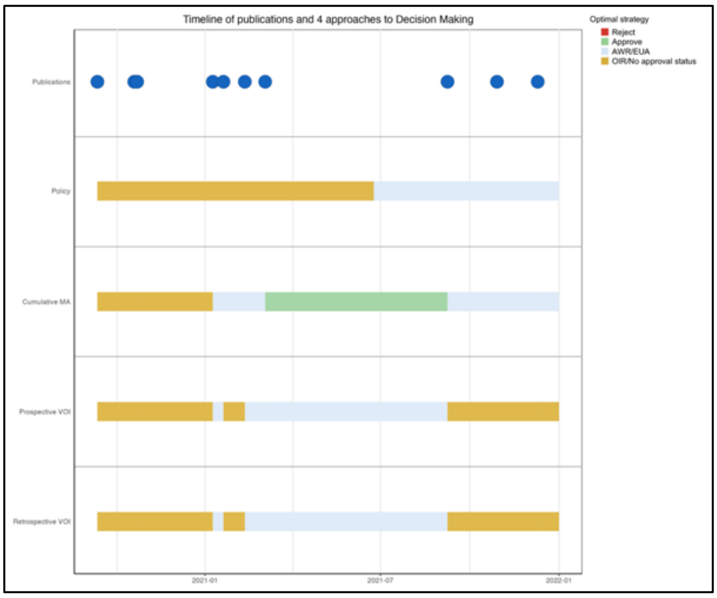

C

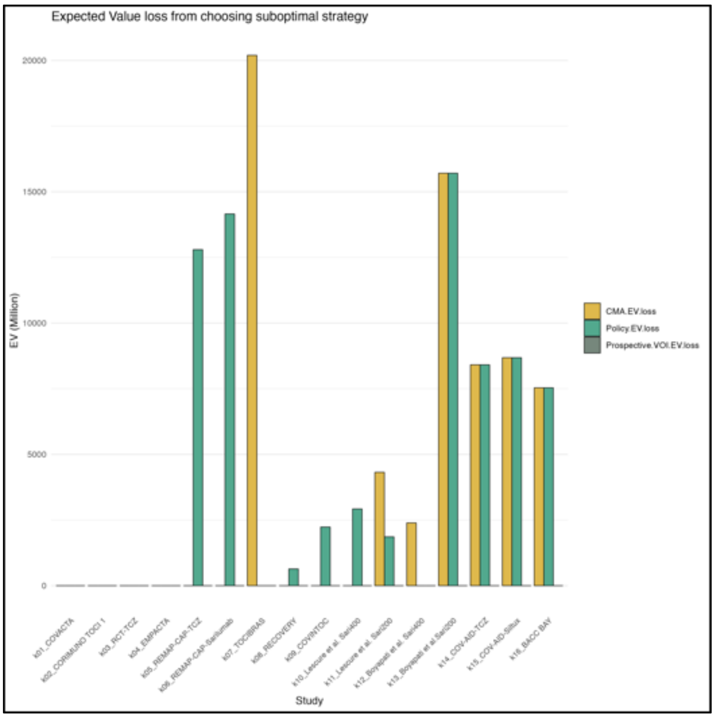

Appendix, Table 11: Sensitivity Analysis – IL-6-(R-)inhibitors

A: Forest plot. The IL-6-(R-)inhibitors subgroup (including those trials investigating Tocilizumab, Sarilumab or Siltuximab) includes 16 papers. Pooled uncertainty is slightly increased compared to the main analysis
B: Timeline results. Approval is no longer suggested at the end of the timeline for CMA.
C: Loss figure comparing approach strategy to retrospective optimal strategy. Depending on the time-point, CMA or Policy approaches lead to most lost.

Reporting guidelines

Appendix, Table 12: PRISMA checklist

Section and Topic	Item #	Checklist item	Location where item is reported
**TITLE**			
Title	1	Identify the report as a systematic review.	N/A as the paper contains multiple methodologies
**ABSTRACT**			
Abstract	2	See the PRISMA 2020 for Abstracts checklist.	N/A as the paper contains multiple methodologies
**INTRODUCTION**			
Rationale	3	Describe the rationale for the review in the context of existing knowledge.	5
Objectives	4	Provide an explicit statement of the objective(s) or question(s) the review addresses.	6
**METHODS**			
Eligibility criteria	5	Specify the inclusion and exclusion criteria for the review and how studies were grouped for the syntheses.	7
Information sources	6	Specify all databases, registers, websites, organisations, reference lists and other sources searched or consulted to identify studies. Specify the date when each source was last searched or consulted.	7
Search strategy	7	Present the full search strategies for all databases, registers and websites, including any filters and limits used.	Appendix Table 1 and 2
Selection process	8	Specify the methods used to decide whether a study met the inclusion criteria of the review, including how many reviewers screened each record and each report retrieved, whether they worked independently, and if applicable, details of automation tools used in the process.	7
Data collection process	9	Specify the methods used to collect data from reports, including how many reviewers collected data from each report, whether they worked independently, any processes for obtaining or confirming data from study investigators, and if applicable, details of automation tools used in the process.	7
Data items	10a	List and define all outcomes for which data were sought. Specify whether all results that were compatible with each outcome domain in each study were sought (e.g. for all measures, time points, analyses), and if not, the methods used to decide which results to collect.	Supplementary file: Appendix
	10b	List and define all other variables for which data were sought (e.g. participant and intervention characteristics, funding sources). Describe any assumptions made about any missing or unclear information.	Supplementary file: Appendix
Study risk of bias assessment	11	Specify the methods used to assess risk of bias in the included studies, including details of the tool(s) used, how many reviewers assessed each study and whether they worked independently, and if applicable, details of automation tools used in the process.	7
Effect measures	12	Specify for each outcome the effect measure(s) (e.g. risk ratio, mean difference) used in the synthesis or presentation of results.	6
Synthesis methods	13a	Describe the processes used to decide which studies were eligible for each synthesis (e.g. tabulating the study intervention characteristics and comparing against the planned groups for	8

Section and Topic	Item #	Checklist item	Location where item is reported
		each synthesis (item #5)).	
	13b	Describe any methods required to prepare the data for presentation or synthesis, such as handling of missing summary statistics, or data conversions.	8
	13c	Describe any methods used to tabulate or visually display results of individual studies and syntheses.	8
	13d	Describe any methods used to synthesize results and provide a rationale for the choice(s). If meta-analysis was performed, describe the model(s), method(s) to identify the presence and extent of statistical heterogeneity, and software package(s) used.	8
	13e	Describe any methods used to explore possible causes of heterogeneity among study results (e.g. subgroup analysis, meta-regression).	8
	13f	Describe any sensitivity analyses conducted to assess robustness of the synthesized results.	8
Reporting bias assessment	14	Describe any methods used to assess risk of bias due to missing results in a synthesis (arising from reporting biases).	7
Certainty assessment	15	Describe any methods used to assess certainty (or confidence) in the body of evidence for an outcome.	7
**RESULTS**			
Study selection	16a	Describe the results of the search and selection process, from the number of records identified in the search to the number of studies included in the review, ideally using a flow diagram.	12, Appendix Figure 1
	16b	Cite studies that might appear to meet the inclusion criteria, but which were excluded, and explain why they were excluded.	NA
Study characteristics	17	Cite each included study and present its characteristics.	Appendix, table 3
Risk of bias in studies	18	Present assessments of risk of bias for each included study.	Appendix, table 4
Results of individual studies	19	For all outcomes, present, for each study: (a) summary statistics for each group (where appropriate) and (b) an effect estimate and its precision (e.g. confidence/credible interval), ideally using structured tables or plots.	Appendix, Table 9
Results of syntheses	20a	For each synthesis, briefly summarise the characteristics and risk of bias among contributing studies.	Appendix, table 4
	20b	Present results of all statistical syntheses conducted. If meta-analysis was done, present for each the summary estimate and its precision (e.g. confidence/credible interval) and measures of statistical heterogeneity. If comparing groups, describe the direction of the effect.	Figure 2
	20c	Present results of all investigations of possible causes of heterogeneity among study results.	Appendix, Figure 6
	20d	Present results of all sensitivity analyses conducted to assess the robustness of the synthesized results.	14; Appendix, Table 7, 9 and 10
Reporting biases	21	Present assessments of risk of bias due to missing results (arising from reporting biases) for each synthesis assessed.	Figure 3
Certainty of evidence	22	Present assessments of certainty (or confidence) in the body of evidence for each outcome assessed.	12
**DISCUSSION**			
Discussion	23a	Provide a general interpretation of the results in the context of other evidence.	15
	23b	Discuss any limitations of the evidence included in the review.	17
	23c	Discuss any limitations of the review processes used.	17

Section and Topic	Item #	Checklist item	Location where item is reported
	23d	Discuss implications of the results for practice, policy, and future research.	19
**OTHER INFORMATION**			
Registration and protocol	24a	Provide registration information for the review, including register name and registration number, or state that the review was not registered.	20
	24b	Indicate where the review protocol can be accessed, or state that a protocol was not prepared.	20
	24c	Describe and explain any amendments to information provided at registration or in the protocol.	NA
Support	25	Describe sources of financial or non-financial support for the review, and the role of the funders or sponsors in the review.	20
Competing interests	26	Declare any competing interests of review authors.	20
Availability of data, code and other materials	27	Report which of the following are publicly available and where they can be found: template data collection forms; data extracted from included studies; data used for all analyses; analytic code; any other materials used in the review.	20

Appendix, Table 13: CHEERS and CHEERS VOI statement

Section and Topic	Item #	Guidance for reporting	Location where item is reported
**TITLE**			
Title	1	Identify the study as an economic evaluation and as a VOI analysis, and specify the interventions being compared.	N/A as the paper contains multiple methodologies
Abstract	2	Provide a structured summary that highlights context, key methods, results, and alternative analyses	3
INTRODUCTION			
Background and objectives	3	Give the context for the study, the study question, and its practical relevance for decision making in policy or practice.	4
METHODS			
Health economic analysis plan	4	Indicate whether a health economic analysis plan was developed and where available.	8
Study population	5	Describe characteristics of the study population (such as age range, demographics, socioeconomic, or clinical characteristics). If population-level VOI measures are estimated, describe and justify how the population who benefits from the research was defined, how the incidence and/or prevalence were derived.	8
Setting and location	6	Provide relevant contextual information that may influence findings.	8
Comparators	7	Describe the interventions or strategies being compared and why chosen.	5
Perspective	8	State the perspective(s) adopted by the study and why chosen.	9
Time horizon	9	State the time horizon for the study assumed in the economic evaluation and why appropriate	9
Discount rate	10	Report the discount rate(s) used in the economic evaluation and for the population-level VOI analysis, and reason chosen.	9
Selection of outcomes	11	Describe what outcomes were used as the measure(s) of benefit(s) and harm(s)	9
Measurement of outcomes	12	Describe how outcomes used to capture benefit(s) and harm(s) were measured	6
Valuation of outcomes	13	Describe the population and methods used to measure and value outcomes.	8
Measurement and valuation of resources and costs	14	Describe how costs were valued.	8
Currency, price date, and conversion	15	Report the dates of the estimated resource quantities and unit costs, plus the currency and year of conversion.	Supplementary file: Excel
Rationale and description of model	16	If modelling is used, describe in detail and why used. Describe the model structure and justify structural assumptions that have been made. Report if the model is publicly available and where it can be accessed.	8
VOI Estimation Methods	S1	(a) Describe the method used to estimate EVPI and any checks used to determine its accuracy. (b) Describe the method used to estimate EVPPI and any checks used to determine its accuracy. (c) Describe the method(s) used to estimate EVSI and any checks used to determine accuracy.	9
Analytics and assumptions	17	Describe any methods for analysing or statistically transforming data, any extrapolation methods, and approaches for validating any model used. Make sure to: (a) State and justify the cost-effectiveness threshold(s) chosen. (b) Describe and justify the statistical and methodological	9

Section and Topic	Item #	Guidance for reporting	Location where item is reported
		choices that were made to estimate parameters (e.g., methods of data synthesis, calibration).	
Evidence base	S2	Discuss the potential risk of bias and heterogeneity in the evidence base and describe whether these were adjusted for in the model.	18
Characterising heterogeneity	18	Describe any methods used for estimating how the results of the study vary for subgroups.	11
Characterising distributional effects	19	Describe how impacts are distributed across different individuals or adjustments made to reflect priority populations.	Supplementary file: Excel
Characterising uncertainty	20	Describe methods to characterise any sources of uncertainty in the analysis. Discuss the key structural uncertainties and how these have been addressed. For parameters subject to a probabilistic analysis, describe how the distributions reflecting uncertainty were derived, including any dependencies between parameters. For parameters that were not subject to a probabilistic analysis, explain why they are considered fixed and known with certainty.	Supplementary file: Excel
Parameters of Interest in VOI analysis	S3	(a) Specify the individual and/or combinations of parameters for which EVPPI has been computed. (b) Specify the individual and/or combinations of parameters for which EVSI has been computed.	9
Study Design(s) proposed in VOI analysis	S4	Describe and justify the design of all proposed future research studies and indicate the model parameters that will be updated by these studies.	9
Data Generation for EVSI	S5	Describe the assumptions used to generate the study data.	9
Costs of Research Studies for ENBS	S6	Provide the costs of research studies proposed in VOI analysis (e.g., fixed costs, variable costs, opportunity costs of allocation), and describe how they were calculated.	Supplementary file: Excel
Approach to engagement with patients and others affected by the study	21	Describe any approaches to engage patients or service recipients, the general public, communities, or stakeholders (such as clinicians or payers) in the design of the study.	Clinicians, Pharmacologists, Health Economists and Methodologists were included in authorship.
RESULTS			
Study parameters	22	Report all analytic inputs (such as values, ranges, references) including uncertainty or distributional assumptions	Supplementary file: Excel
Summary of main results	23	Report the mean values for the main categories of costs and outcomes of interest and summarise them in the most appropriate overall measure. Report the values for the VOI measures considered	Table 1
Effect of uncertainty	24	Describe how uncertainty about analytic judgments, inputs, or projections affect findings. Report the effect of choice of discount rate and time horizon, if applicable.	Figure 3
Effect of engagement with patients and others affected by the study	24	Report on any difference patient/service recipient, general public, community, or stakeholder involvement made to the approach or findings of the study.	Choice in main and sensitivity analyses, prioritization of outcomes,
DISCUSSION			
Study findings, limitations, generalisability, and current knowledge	26	Report key findings, limitations, ethical or equity considerations not captured, and how these could affect patients, policy, or practice.	15
OTHER RELEVANT			

Section and Topic	Item #	Guidance for reporting	Location where item is reported
INFORMATION			
Sources of funding	27	Describe how the study was funded and any role of the funder in the identification, design, conduct, and reporting of the analysis	20
Conflicts of interest	28	Report authors conflicts of interest according to journal or International Committee of Medical Journal Editors requirements.	21

ENBS, expected net benefit of sampling; EVPI, expected value of perfect information; EVPPI, expected value of partial perfect information; EVSI, expected value of sample information. The new items introduced in the CHEERS VOI checklist compared to the checklist provided by the CHEERS statement are numbered starting with 1 and proceeded with letter ‘S.’
